# 3D printed anisotropic tissue simulants with embedded fluid capsules for medical simulation and training

**DOI:** 10.1126/sciadv.adw6446

**Published:** 2025-08-29

**Authors:** Adarsh Somayaji, Matthew S. Lawler, Alex T. Gong, Zachary J. Fuenning, Victoria A. Roach, Athira B. S., David J. Traina, Jason R. Speich, Ruikang K. Wang, Matthew G. Hackett, David M. Hananel, Robert M. Sweet, Michael C. McAlpine

**Affiliations:** ^1^ Department of Mechanical Engineering, University of Minnesota, Minneapolis, MN 55455, USA.; ^2^ Department of Biomedical Engineering, University of Minnesota, Minneapolis, MN 55455, USA.; ^3^ Department of Surgery, University of Washington, Seattle, WA 98195, USA.; ^4^ Department of Bioengineering, University of Washington, Seattle, WA 98195, USA.; ^5^ Department of Ophthalmology, University of Washington, Seattle, WA 98105, USA.; ^6^ Army Research Laboratory, Orlando, FL 32826, USA.

## Abstract

Human tissues are primarily composed of collagen and elastin fiber networks that exhibit directional mechanical properties that are not replicable by conventional tissue simulants manufactured via casting. Here, we 3D print tissue simulants that incorporate anisotropic mechanical properties through the manipulation of infill voxel shape and dimensions. A mathematical model for predicting the anisotropy of single- and multimaterial structures with orthogonal infill patterns is developed. We apply this methodology to generate conformal printing toolpaths for replicating the structure and directional mechanics observed in native tissue within 3D printed tissue simulants. Further, a method to embed fluid-filled capsules within the infill structure of these tissue simulants to mimic blood is also presented. The improvements in simulation quality when using 3D printed anisotropic tissue simulants over conventional tissue simulants are demonstrated via a comparative acceptability study. These advances open avenues for the manufacture of next-generation tissue simulants with high mechanical fidelity for enhanced medical simulation and training.

## Teaser

Advanced 3D printed simulants with embedded fluid capsules mimic real tissue mechanics, providing more realistic medical training.

## INTRODUCTION

Tissue simulation involves the replication of a real-world medical scenario to achieve specific training objectives (*1*). The use of synthetic organ models in medical training and education dates back centuries and was intended to help trainees learn technical skills in a low-risk environment (*2*). Tissue and organ simulants attempt to model specific features of the human body and have different degrees of accuracy at which the anatomical, physiological, and mechanical properties are represented (*3*, *4*). In recent years, there has been increased interest in improving the fidelity of physical organ simulants, with research showing that these simulants provide valid learning experiences and help novice and experienced trainees in developing enhanced motor skills and decision-making capabilities (*5*, *6*). Medical simulators also improve surgical outcomes by increasing the ability of surgeons to complete procedures, decreasing operation times, and lowering error and complication rates (*7*–*9*). While conventional physical tissue simulants are manufactured by methods such as casting, advances in three-dimensional (3D) printing technologies have led to the development of customizable, structurally accurate, and patient-specific organ models (*10*–*14*).

Despite these advances, gaps remain in truly replicating the mechanical behavior exhibited by biological tissue within tissue simulants. A major source of interest in studying the simulation of biological tissues is that tissues exhibit substantial directional anisotropy and nonlinearity in mechanical properties (*15*). This behavior is manifested differently in different types of tissues due to the unique composition of biological tissues wherein an extracellular matrix composed of collagen, elastin, and other proteins form a network of interconnected fibrous elements. When subjected to deformation, these biocomposite fiber networks show a nonlinear response characterized by an increased stiffness with increasing strain (*16*). The particular arrangement of these fibers within the matrix also generates a directional anisotropy in their mechanics, where the stiffness of tissue along the direction of dominant collagen fiber orientations is higher than the stiffness in the direction normal to this orientation (*17*–*20*). Computational models of human tissues have demonstrated the importance of anisotropic behavior in providing directional strength as well as flexibility (*21*). Models that incorporate anisotropy exhibit lowered stresses in response to the same applied deformations.

Human tissues exhibit a wide range of mechanical anisotropy with variations of the elasticities in orthogonal directions up to 6:1 for skin tissue in certain locations (*17*). Previously, researchers have developed numerical optimization techniques to generate tiled microstructures that can be built up using selective laser sintering (SLS) to manufacture objects with anisotropic mechanical properties (*22*). However, SLS is better suited for the manufacturing of stiff or semirigid parts, and as such is not ideal for tissue simulation applications. Furthermore, SLS generally supports only a single input material, limiting the range of attainable anisotropy with this approach to about 2.5:1. Recently, researchers have included anisotropic mechanics in tissue simulants by using a fiber-matrix model in which stiff fibers are embedded into a matrix via molding (*23*, *24*). While this method improves the mechanical fidelity of synthetic tissue simulants, the resultant simulants had low levels of anisotropy (~1.2:1). Therefore, this method is not well suited to the manufacturing of complex-shaped organ models and simulants with high anisotropy. Other publications using 3D printing to introduce anisotropic mechanics into structures relied on stiff fillers embedded in a soft matrix. While these researchers were able to demonstrate a resultant mechanical anisotropy of ~6:1, the addition of fibers greatly increased the elastic modulus of the composite structure (*25*). To the best of our knowledge, a method that can independently tune elastic moduli along orthogonal directions, enabling precise control over both the elasticity and the anisotropy in mechanical response, has not been previously described in literature.

Here, we propose a methodology for incorporating the anisotropic mechanics of human tissue in synthetic tissue simulants. We exploit the inherent anisotropy that is characteristically observed in extrusion-based 3D printing processes to design and control the mechanical behavior of tissue simulants. We selected a surgical cricothyrotomy (cric) training procedure as a reference case for developing our methodology. A cricothyrotomy is a lifesaving procedure that is performed in situations where conventional airway management techniques fail to restore effective ventilation to a patient with respiratory distress (*26*). In such “cannot-intubate, cannot-oxygenate” (CICO) situations, the cricothyroid membrane, a thin ligament in the neck that connects the thyroid and cricoid cartilages, is punctured using a scalpel and an intubation tube is inserted into this incision to restore ventilation (*27*). The incidence rates of CICO situations range from 0.2 to 0.3% (*28*) in hospital emergency rooms to 12% (*29*) in prehospital combat settings.

Because this procedure is only performed as a last resort, it cannot be taught in live situations, and most clinicians only receive theoretical training in the techniques involved (*30*). The development of medical simulators provides an avenue for using animal tissue (*31*) or synthetic skin (*32*) as an analog while training for surgical cricothyrotomies. Studies have shown that animal tissue is superior to synthetic skin models in training cricothyrotomies due to its higher anthropomorphic properties and tissue fidelity (*33*). Cadaver-based training has been reported to be superior to simulation training as it naturally incorporates anatomical variations in landmarks, tissue structures, and cavities (*34*). Nevertheless, cadavers are expensive and nonreusable, and most clinical settings have limited access to them. Animal-derived tissue is also difficult to obtain and store. Therefore, a method to enhance the mechanical fidelity of synthetic skin simulants is critical to improving training standards and upskilling clinicians.

We developed a mathematical model that relates the dimensions of print-line parameters such as print-line height and print-line spacing to the mechanical anisotropy of the 3D printed structure. We then developed ink formulations for synthetic tissues that can mimic the elastic moduli of human tissue. Moreover, we demonstrated techniques to adjust the printing parameters to enable the deposition of the desired voxel structure on nonplanar surfaces using a standard three-axis linear positioning system. We integrated these techniques into a slicing and toolpathing algorithm that combines tissue shape, the underlying collagen fiber orientations, and mechanical anisotropy to determine the printing toolpaths for manufacturing simulants with high mechanical fidelity.

The presence of blood in the simulation is an important factor in enhancing procedural realism (*35*, *36*). To simulate bleeding, the current process of manufacturing tissue simulants involves the manual injection of simulated blood at specific locations before sealing. A method to automate the storage of fluids within the tissue simulants can further streamline the process of manufacturing these simulants, particularly if the fluid storage locations are difficult to access. To address this issue, we studied the effectiveness of water-oil-water (W-O-W) double-emulsion (DE) capsules for storing simulated blood within the tissue simulants. The creation of DE capsules enables the separation of liquid payloads such as blood, pus, and serous fluid from the structural tissue layers of the simulant to prevent interference during the curing process. Last, we evaluated the performance of 3D printed simulants through a comparative acceptability study to the current state-of-the-art simulants manufactured by casting.

Our developed approach uses a conventional gantry-based 3D printer to achieve highly accurate deposition characteristics while not substantially affecting 3D printing process parameters such as printing time, resolution, and material handling complexity. These techniques are generalizable and can be used to 3D print laminar organ models with the desired mechanical anisotropies and directionalities. We anticipate that these tools can help bridge the gap in realism and mechanical fidelity between tissue simulants and real human tissue, thereby improving the quality of medical training and education as well as surgical outcomes.

## RESULTS

### Cricothyrotomy training puck

**Fig. 1.**
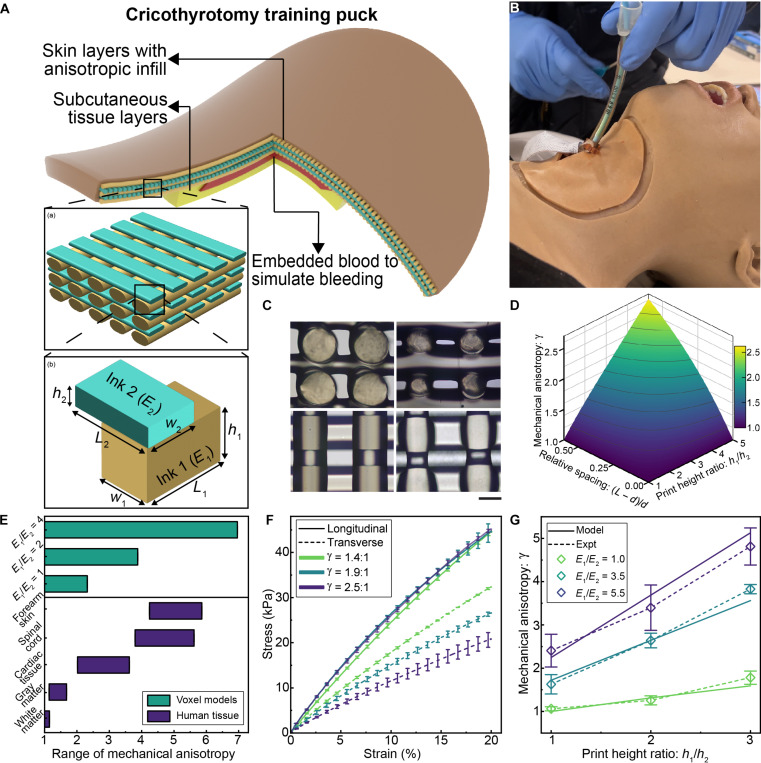
Design and characterization of cellular voxel models. (**A**) Typical structure of a 3D printed anisotropic cricothyrotomy puck for surgical training. The skin layers contain an anisotropic infill pattern sandwiched by top and bottom cover layers. (a) A typical infill pattern that generates mechanical anisotropy. (b) The simplest voxel structure with variables labeled. (**B**) Image of a cricothyrotomy puck mounted on an advanced airway trainer (CREST Lab, University of Washington) for training emergency cricothyrotomies. (**C**) Microscope images of the cross section of the infill pattern along orthogonal directions (top). Microscope images of the infill patterns as seen from above (bottom) (scale bar, 500 μm). (**D**) Variation of anisotropy with underlying infill parameters. The parameter space is reduced by setting the spacing between lines across successive layers to be equal. (**E**) Range of mechanical anisotropies that are observed in the human body (bottom) and achievable using various material combinations (top). (**F**) Tensile testing plots of 3D printed anisotropic cruciform-shaped samples in the axial and transverse directions (*n* = 3, error bars indicate SD). These samples were printed with a single material (i.e., $E_{1}=E_{2}$ ). The plot shows that different values of mechanical anisotropy can be achieved by simply modifying the underlying voxel dimensions. (**G**) Plot showing the correspondence between predicted and experimental values of mechanical anisotropy of infill structures 3D printed with two materials along alternate directions (*n* = 3, error bars indicate SD). To reduce dimensionality, the spacing between print lines was kept constant and the ratio of print heights between the stiffer and softer materials were varied across samples.

The Advanced Joint Airway Management System (AJAMS) cricothyrotomy training puck (cric-skin puck) is a single-use disposable tissue model that has a hyperbolic paraboloid shape and consists of skin and subcutaneous tissue layers separated by a thin cavity into which simulated blood is filled (Fig. 1A). The thicknesses of the skin layer and subcutaneous tissue layers are 2.75 and 3.25 mm, respectively. Commercially available surgical tape has been shown to accurately replicate the required 1 N of puncture force and is, therefore, used to simulate the cricothyroid membrane (*37*).

Extrusion-based 3D printing processes involve the deposition of materials layer-by-layer to form a structure. To incorporate anisotropic mechanics, the skin layer of the cric-skin puck was designed to have a cellular infill structure that is composed of a grid of overlapping orthogonal print lines in which each layer spans the gaps between lines of the previous layer to form a periodic lattice [Fig. 1A (a)] (*38*, *39*). This structure can be represented as a repetitive arrangement of a single orthogonal unit of print lines, hereby referred to as a voxel of material [Fig. 1A (b)]. The subcutaneous tissue layers were printed with full infill and, therefore, no anisotropy. To train medical professionals in performing this procedure, the cric-skin pucks are overlaid on an advanced airway management manikin (*40*) that can reproduce the breathing mechanics of a person (Fig. 1B).

### Voxel model development

The mechanical behavior of the superstructure composed of the voxel units described above can be understood by modeling the behavior of the underlying voxel unit under deformation as described in the equations below (fig. S1 and supplement 1). While the filaments have a circular cross section due to the use of a cylindrical nozzle, we assume a rectangular cross section to simplify the mathematical model. The anisotropic ratio γ is defined as the ratio between the apparent longitudinal modulus $E_{L}$ and the apparent transverse modulus $E_{T}$

$$E_{L}=\frac{\left\{V_{L1}*E_{1}*\left[V_{L1}*E_{1}+\left(V_{S2}+V_{L2}\right)*E_{2}\right]\right\}}{\left[V_{B}*\left(V_{L1}*E_{1}+V_{S2}*E_{2}\right)\right]}$$ (1)

$$E_{T}=\frac{\left\{V_{L2}*E_{2}*\left[V_{L2}*E_{2}+\left(V_{S1}+V_{L1}\right)*E_{1}\right]\right\}}{\left[V_{B}*\left(V_{L2}*E_{2}+V_{S1}*E_{1}\right)\right]}$$ (2)

$$\gamma =\frac{E_{L}}{E_{T}}$$ (3)

where $\begin{array}{c} \;V_{L1}=w_{1}h_{1}L_{1},V_{L2}=w_{2}h_{2}L_{2},V_{S1}=L_{1}h_{1}\left(L_{2}-w_{1}\right),V_{S2}=L_{2}h_{2}, \end{array}$ $\begin{array}{c} \left(L_{1}-w_{2}\right),\text{and}\;V_{B}=L_{2}\left(h_{1}+h_{2}\right) \end{array}$ are computed from the voxel microdimensions.

A microscopic view of a typical infill structure shows the internal arrangement of print lines within (Fig. 1C). To reduce the parameter space, the print-line widths $\left(w_{1}\;\text{and}\;w_{2}\right)$ were set to be equal to the nozzle diameter (d) by controlling the extrusion flow rate via an endless piston–based volumetric dosing pump, while the print heights $\left(h_{1}\;\text{and}\;h_{2}\right)$ , print-line spacings $\left(L_{2}-d\;\text{and}\;L_{1}-d\right)$ , and constitutive materials $\left(E_{1}\;\text{and}\;E_{2}\right)$ were assigned as the primary variables. If we set the constitutive materials to be identical (i.e., $E_{1}=E_{2}$ ), then the mechanical anisotropy expressed by these structures can simply be increased by increasing the ratio of print heights or by increasing the print-line spacing (Fig. 1D). However, the material extrusion process places certain practical limits on the range of values of each voxel parameter. For instance, if the print height were to be substantially increased, then the extruded filament would fail to adhere to the substrate and would instead curl around the nozzle tip. Similarly, if the print-line spacings were substantially increased, then the print lines would fail to span the distance (*41*). Therefore, the range of mechanical anisotropy that can be achieved with given input materials is bounded by these limits. This range can be increased by increasing the ratio of elastic moduli of the constituent materials, thereby enabling this voxel design to be compatible with the anisotropic properties of human tissue (Fig. 1E) (*18*, *19*, *42*).

Typical stress-strain curves of anisotropic samples printed with different anisotropic ratios demonstrate the capability of this methodology to generate structures with similar mechanical responses in the longitudinal directions but different levels of responses in the transverse directions (Fig. 1F). To test the accuracy of the model, cruciform-shaped samples consisting of six anisotropic infill layers and two fully filled isotropic cover layers were 3D printed with an 18-gauge (GA) nozzle (*d* = 840 μm) and subjected to tensile testing. The print-line spacings $\left(L_{2}-d\;\text{and}\;L_{1}-d\right)$ for the infill layers were kept constant (i.e., $L_{2}-d=L_{1}-d$ = 300 μm), while the constitutive materials and print heights for alternate layers were varied. The tensile test results show that the measured values of mechanical anisotropy are close to the mechanical anisotropy predicted by the model across cases, showcasing the validity of the model (Fig. 1G and fig. S2). Because a cellular infill structure inherently contains gaps, the tactile response to scalpel indentation and puncture may vary between overlapping and spanning sections. To evaluate this, we used an experimental setup capable of measuring puncture force profiles during scalpel penetration through tissue samples (*37*). Our results showed no notable variation in response with respect to the direction of puncture (i.e., longitudinal or transverse directions) or between overlapping and spanning sections (fig. S3).

### Material selection and characterization

The direct-ink-write 3D printing process involves the extrusion through a nozzle of a shear-thinning viscoelastic material that is subsequently cured to obtain a solidified structure. The formulation of the ink plays a key role in determining the mechanical performance of the tissue simulant. It is desirable to have a formulation that can be adjusted such that inks with a wide range of postcure elastic moduli can be extruded with near-identical rheological properties. Previously, we developed silicone-based polymeric inks that mimic the elastic properties of soft tissue such as the prostate and aortic valve leaflets (*12*–*14*). These inks consisted of a room temperature vulcanizing silicone (RTVS) mixed with silicone grease as a bulking agent to adjust the elastic properties of the cured product. While these inks could be used to print structures with full infill, the 3D printing of cellular structures proved to be a challenge as inks with reduced storage moduli would not be able to span the gaps created between successive print lines within cellular infill structures (*43*).

**Fig. 2.**
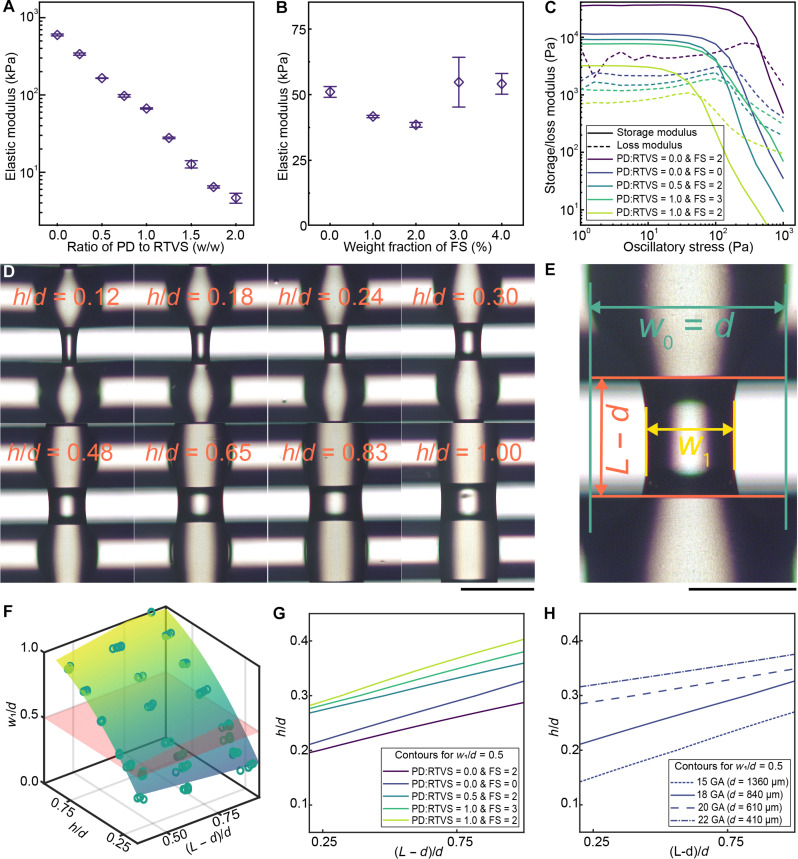
Material selection and optimization for 3D printing cellular structures. (**A**) The variation in the elastic moduli of cured mixtures of PlatSil Deadener (PD) and room temperature vulcanizing silicone (RTVS) with a fixed amount of fumed silica (FS) (*n* = 3, error bars indicate SD). The elastic modulus can be adjusted by two orders of magnitude. (**B**) The variation in the elastic moduli of cured mixtures of a fixed ratio of PD and RTVS with varying amounts of FS (*n* = 3, error bars indicate SD). The elastic modulus does not vary substantially. (**C**) The variation in the storage and loss moduli of uncured PD/RTVS/FS inks. By adjusting the amounts of PD and FS, we can control ink rheology. For instance, inks PD:RTVS = 0.0 and FS = 0, PD:RTVS = 0.5 and FS = 2, and PD:RTVS = 1.0 and FS = 3 show similar rheology. (**D**) Variation in print-line width of deposited material spanning gaps in the previous layer due to cohesive forces as seen through a microscope (scale bar, 500 μm). (**E**) Micrograph of a singular spanning print-line with measured dimensions labeled (scale bar, 250 μm). (**F**) The relative reduction in print-line width at different relative print heights and print-line spacings for a particular ink. The print-line width reduction is particularly sensitive to a reduction in print height. A cutting plane at $w_{1}/d$ = 0.5 is also shown. (**G**) Contour plots of $w_{1}/d$ = 0.5 plotted for inks with different storage moduli. Increasing storage moduli results in lowered bounds for print height and greater obtainable ranges of mechanical anisotropy. All samples printed with an 18-GA nozzle. (**H**) Contour plots of $w_{1}/d$ = 0.5 plotted at different values of *d*. Increasing nozzle diameter results in lowered bounds for print height and greater obtainable ranges of mechanical anisotropy. All samples printed with PD/RTVS = 0.0 and FS = 0 ink.

We replaced the bulking agent used in our previous research (*14*) with a low-viscosity PlatSil Deadener (PD) that showed a substantial improvement in the range of elastic moduli that could be obtained (Fig. 2A). By adjusting the weight ratio of PD to RTVS from 0.0 to 2.0, we were able to vary the elastic modulus of the cured inks by two orders of magnitude (from ~5 to ~600 kPa). The addition of PD to RTVS beyond a weight ratio of 1:1 results in increased tackiness; therefore, the cover layers needed to be printed with a lower PD:RTVS ratio or a small amount of talc needed to be applied onto the cover layers. Additionally, increasing the PD:RTVS ratio results in lowering the viscosity of the mixture. This is compensated by adding fumed silica (FS) as a thickening agent. The addition of FS does not substantially change the elastic modulus of the cured material (Fig. 2B) while allowing the modification of the rheological characteristics of the inks such that they have near-identical rheological behaviors under shear (Fig. 2C and fig. S4, A and B). For instance, inks PD:RTVS = 0.0 and FS = 0, PD:RTVS = 0.5 and FS = 2, and PD:RTVS = 1.0 and FS = 3 showed similar rheological behaviors while exhibiting postcure elastic moduli of ~561, ~165, and ~ 55 kPa, respectively. As FS has a negative zeta potential, a small quantity of fumed alumina (FA), which exhibits a positive zeta potential, was used to prevent electrostatic charging of inks during extrusion with the endless piston (fig. S4C). Last, coloring pigments were used to match the color of the tissue simulant to the color of the manikin.

When overlapping lines are deposited in the cellular form as described by the anisotropic voxel model, a narrowing of the section spanning the spacing between lines in the previous layer was observed due to the cohesive forces of attraction between silicone molecules (Fig. 2D). The narrowing of the spanning section is detrimental to the strength of the cellular structure and results in deviations from the predicted anisotropy from the mathematical model. We measured the relative reduction of the width of the spanning section $\frac{w_{1}}{w_{0}}$ for different ink formulations and various combinations of print heights and print-line spacings using a microscope (Fig. 2E). Pure RTVS with no additives was used as the ink formulation for the lower layer to ensure identical spanning conditions for each test formulation. For a particular formulation, the relative width reduction plotted as a function of the print height and print-line spacing, normalized with respect to the nozzle diameter, showed that, as the print height is lowered and the print-line spacing is increased, the width of the spanning section is reduced (Fig. 2F). The relative width reduction is particularly prominent at lower print heights where the surface area–to–volume ratio of the deposited material is higher. Aside from the printing parameters, we found that the reduction in print-line width when spanning a gap was primarily a function of the storage moduli of the silicone-based inks, where inks with a higher storage modulus showed a lower reduction in print-line width under identical conditions. This is visualized by plotting the bounds of print height and print-line spacing at which the reduction in print-line width is equal to 50% for various inks (Fig. 2G). These bounds are used to determine the limits of the printing parameters and, consequently, the limits of the anisotropy for a particular choice of ink. We also studied the effect of using smaller or larger nozzle sizes for a particular ink to determine the scalability of the voxel structure (Fig. 2H). As expected, larger nozzles showed a greater range of $\frac{h}{d}$ values for which the width reduction is ≤50%. This shows that the physical thickness of the print layer is a primary determinant of the effect of the cohesive forces. Therefore, tissue simulants that are 3D printed with smaller-sized nozzles would have a diminished range of achievable anisotropy compared to larger nozzles.

### Deposition on nonplanar surfaces

Organs in the human body typically do not have a well-defined geometric shape. The cric-skin puck used for cricothyrotomy training has a hyperbolic paraboloid shape with varying degrees of curvature at different locations. To obtain predictable anisotropic mechanical behavior at all locations within the cric-skin puck, the cellular voxel structure must be oriented along the natural topography of the organ model. Therefore, the ability to 3D print anisotropic cric-skin pucks is predicated on the accuracy of deposition on curved surfaces.

**Fig. 3.**
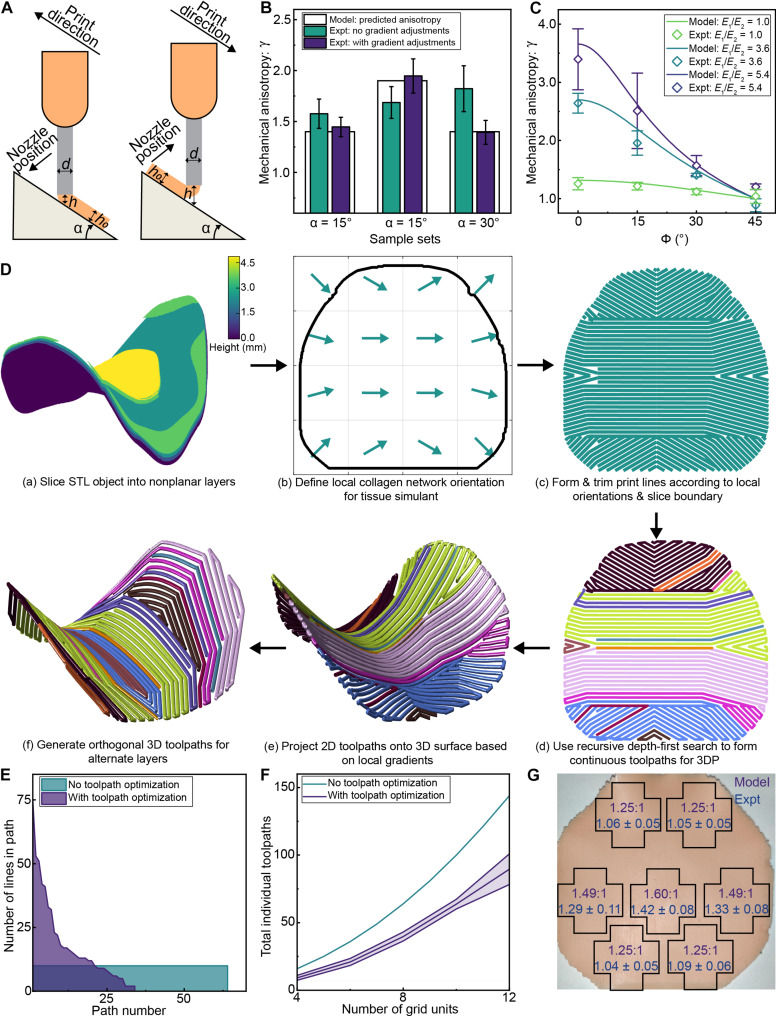
Incorporation of directional anisotropy in nonplanar tissue simulants. (**A**) Deposition accuracy on inclined surfaces can be improved by adjusting nozzle position relative to substrate in accordance with ink rheology, angle of inclination and gradient direction. (**B**) Incorporation of nozzle adjustments led to improved accuracy of deposition on inclined surfaces and improved the accuracy of measured values of anisotropy to predicted values (*n* = 3). (**C**) The variation in mechanical anisotropy when the relative angle between the direction of maximum anisotropy and the measurement axes is varied (*n* = 3). The measured values of anisotropy closely match the values of anisotropy predicted by the mathematical model. (**D**) Process of 3D printing a tissue simulant in accordance with its physical structure and local variations in collagen fiber orientations. (**E**) The number of waypoints in each individual toolpath is plotted against the path number before and after toolpath optimization for an 8 × 8 grid. Following optimization, paths that are longer and continuous are preferentially generated to reduce the total number of start-stop extrusion events. (**F**) The number of individual toolpaths with and without optimization is plotted against the number of grid units. The spread indicates SD (*n* = 5). The toolpathing algorithm provides a reduction of ~40% in the total number of toolpaths regardless of the number of grid units defined. (**G**) The local variation in anisotropy of samples 3D printed with the directionality as described above is shown (*n* = 3). The predicted anisotropy is shown on top while the measured anisotropy is shown below it. Each sample shows a consistent reduction of measured anisotropy relative to the predicted value due to the presence of thick cover layers. Yet, the local variation of anisotropy is retained, demonstrating the utility of this process in generating structures with locally defined mechanics.

In previous research, increasing the degrees of freedom (DOFs) by using 6-DOF industrial robots (*44*, *45*) or rotatory build platforms (*46*) served as a method to directly align the dispensing tip to the local surface normal at each point to enable conformal 3D printing. While having additional DOFs could help improve deposition accuracy, the implementation of such systems presents certain challenges. For instance, achieving highly accurate voxel-scale dimensions that are critical for generating predictable anisotropic mechanics requires the maintenance of constant motion speed and extrusion rates. However, enforcing a strict 6D pose in addition to a constant speed constraint can lead to infeasible configurations, causing the manipulator to slow down or pause to avoid violating joint limits that would, in turn, negatively affect deposition fidelity. Additionally, robotic manipulators often have limited payload capacity, rendering them less suitable for applications involving multiple flow control systems and extruders. Other researchers developed methods to adapt to a nonplanar substrate with a conventional Cartesian gantry positioning system by adjusting the print height based on the local inclination of the substrate (*47*, *48*). On the basis of the methods discussed above, we found that the deposition accuracy on nonplanar substrates can be improved by adjusting the print-line height based on the local gradient of the substrate (Fig. 3A) as follows

$$h=\frac{h_{0}}{\text{cos}\;\alpha \;}\pm \left(\frac{d}{2}\right)*\text{tan}\;\alpha$$ (4)

where *h* is the adjusted print-line height, *h*_0_ is the desired layer height, α is the local angle of inclination, and *d* is the nozzle inner diameter (supplement 2). Performing these adjustments led to improved accuracy in the measured mechanical anisotropy when 3D printing cellular voxel structures on a plane with a fixed angle of inclination (Fig. 3B and fig. S5). The cross-sectional views of the skin layer of a 3D printed cric-skin puck demonstrate the utility of this method in achieving high deposition quality (fig. S6).

### Development of toolpathing algorithms for directional anisotropy

In addition to the voxel parameters, the variation in mechanical anisotropy is dependent on the relative orientation (ϕ) between the measurement axes and the orthogonal print-line directions. If the print-line spacings for alternate layers are kept equal, then this variation can be modeled as described below (supplement 3 and fig. S7).

$$\gamma \left(\phi \right)=\frac{\sqrt{E_{L}^{2}*{\text{cos}}^{2}\left(\phi \right)+E_{T}^{2}*{\text{sin}}^{2}\left(\phi \right)\;}}{\sqrt{E_{L}^{2}*{\text{sin}}^{2}\left(\phi \right)+E_{T}^{2}*{\text{cos}}^{2}\left(\phi \right)\;}}$$ (5)

The accuracy of the model in predicting the anisotropy at different relative orientations of the measurement axes was tested by 3D printing cruciform-shaped samples with different ϕ and subjecting the samples to tensile testing. The measured anisotropy for each sample set showed good agreement with the predictions from the model (Fig. 3C).

### Processing of STL models

A stereolithography (STL) model of the cric-skin puck was imported into MATLAB and reoriented such that the overall height of the model was minimized [Fig. 3D (a)]. The bottom and top layers of the model were separated by computing the surface normal at each triangulation vertex and segregating the vertices based on the direction of the *Z*-component of the surface normals. The nonplanar bottom layer was used as a template, and the STL model was successively sliced in accordance with the shape of the bottom layer and desired model anisotropy parameters using a custom MATLAB script. The model was defined to have an infill structure based on the chosen voxel parameters sandwiched by a top and bottom cover layer with full infill. To 3D print the cric-skin puck, a reusable deposition substrate in the shape of the bottom layer was manufactured on a STL-based 3D printer.

### Identification of underlying collagen fiber network orientations

The intrinsic optical anisotropy of fibrous collagen allows for high-contrast mapping of axis orientation, offering valuable insights into collagen alignment within tissue (*49*–*51*). We used polarization-sensitive optical coherence tomography (PSOCT) (*52*, *53*) to image the local orientations of the collagen fiber network of skin tissue in the neck region. The field of view of the system is ~10 mm by 10 mm, and, to capture the collagen organization in the marked ~4 cm by 4 cm region, we conducted 16 measurements, each covering ~10 mm by 10 mm (fig. S8A). Computational stitching was used to integrate the results, referencing different paper cutouts placed on the skin surface. The stitched local en face projection of the optics axis (within a 200- to 225-μm slab from the skin surface) was derived from the PSOCT measurement (fig. S8B). This axis represents the anisotropy axis of the collagen, indicating the organization of collagen in the dermal region of the skin. The results clearly show a horizontal organization of collagen, consistent with Langer’s lines (*54*).

### Toolpath optimization for directional anisotropy

Toolpaths for printing each layer were generated on the basis of the chosen underlying voxel model and anisotropy parameters. We propose the use of locally orthogonal print lines to mimic local collagen fiber orientations as a method to replicate the macroscale mechanics of the tissue. Using MATLAB, each 3D printing slice was discretized into a grid with the primary collagen fiber orientation for each grid unit defined using Langer’s lines [Fig. 3D (b)]. To promote the formation of continuous print lines, the program generated print lines in each unit successively such that the points located on the border in previous units acted as potential starting points for subsequent units. Separate cases were written to resolve issues such as an unequal number of print lines in neighboring grid units to obtain smooth continuous print lines. The contour of each layer was generated and overlaid on top of the generated grid to trim points that lie outside the layer borders [Fig. 3D (c)].

Next, the generated print lines are to be traversed in a manner that minimizes the number of stop-start extrusion events. A recursive depth-first search algorithm performed this optimization: (i) generate a random seed point from the set of nontraversed way points (seed pool); (ii) propagate forward from the selected point until a branching point is reached; (iii) explore each branch using a recursive depth-first search and choose the longest possible path; (iv) repeat the process in the reverse direction; (v) once the longest continuous path is found, remove traversed way points from the seed pool; (vi) repeat the process until all points from the seed pool are accounted for.

The optimization algorithm was executed for many iterations with different seed points and the set with the least number of distinct paths was chosen for 3D printing [Fig. 3D (d)]. Following the identification of 2D toolpaths, the 3D curves for each segment were computed by projecting the 2D toolpath onto the 3D surface [Fig. 3D (e and f)]. The numerical gradient was computed for each curve and the nozzle height was adjusted on the basis of the angle of the gradient. Last, a text file containing machine commands for our custom 3D printing system was outputted from the MATLAB script.

To compare the number and length of distinct paths before and after toolpath optimization, an 8 × 8 grid with random local orientations was generated and waypoints were generated such that each grid unit had 10 individual waypoints. Without toolpath optimization, each grid unit can be filled in separately, resulting in 64 distinct paths with 10 waypoints each. Following toolpath optimization, the number of waypoints in some individual paths substantially increased, resulting in improved printing continuity and an overall reduction in the total number of distinct paths (Fig. 3E). On average, ~80% of the printing area was traversed with more continuous toolpaths after optimization. The application of the toolpath optimization algorithm reduced the total number of individual paths by ~40% regardless of grid size (Fig. 3F).

A cric-skin puck was 3D printed with the directional variation of anisotropy shown in Fig. 3D (movie S1). Cruciform-shaped samples were extracted from various locations and subjected to tensile testing. The predicted and measured values of mechanical anisotropy at each location closely matched (Fig. 3G). In all cases, the mean measured value of anisotropy was lower than the predicted value as the printing substrate contains gradients of ~45°, which necessitated an increase in the thickness of the cover layer to prevent nozzle-substrate interference. This method can be also applied to organ models of smaller scales such as the aortic valve (fig. S9 and movie S2). Thus, this process can generate a 3D printed tissue simulant with localized variations in mechanical anisotropy to mimic the natural variations in mechanics observed in human tissue.

### Integration of fluid-filled capsules within cric-skin puck

Fluids such as simulated blood or pus can be integrated into tissue simulants to further recapitulate native tissue behavior. The current manufacturing standard is to create cavities that are injected with simulated fluids postcuring. Fluid introduction can be automated by incorporating this step into the tissue simulant 3D printing process. To 3D print fluids into the tissue simulants and ensure that the integrated fluids do not flow or interfere with the curing process, we created submillimeter-scale DE microcapsules (*55*) to encase the simulated fluids until required for medical training.

**Fig. 4.**
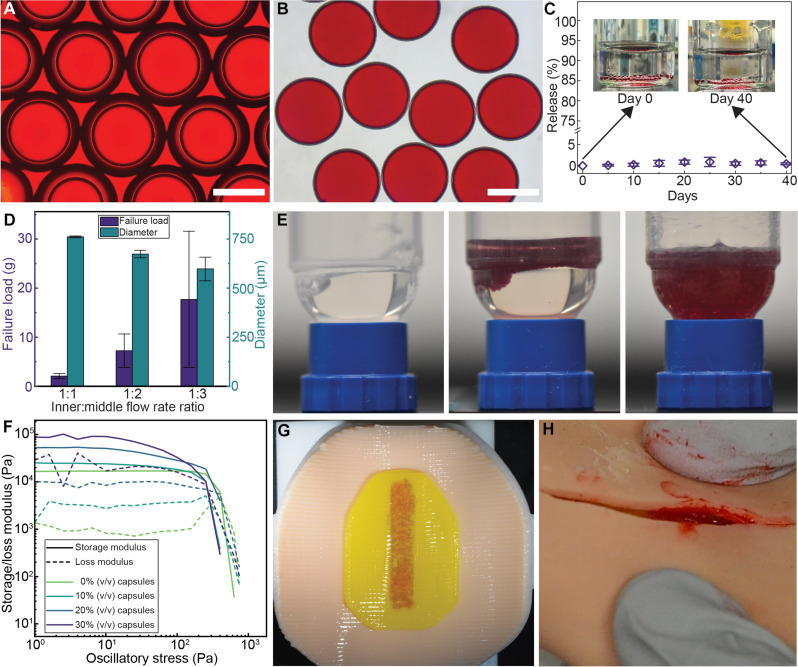
Incorporation of fluid-filled capsules to simulate bleeding. (**A**) Micrograph of double-emulsion capsules immediately after production and before evaporation of shell solvent (scale bar, 500 μm). (**B**) Micrograph of capsules after evaporation of shell solvent showing consistent sizing (scale bar, 500 μm). (**C**) Capsules show minimal passive release of their core contents when stored in an osmotically balanced collecting solution (*n* = 3, error bars indicate SD). Inset: Pictures of 120 capsules stored in collecting solution at day 0 and day 40. The collecting solution shows minimal discoloration. (**D**) The capsule shell properties such as size and maximum force required for failure can be customized by adjusting the relative flow rates of the inner phase and middle phase during capsule production (*n* = 7, error bars indicate SD). (**E**) The process of embedding capsules into a sacrificial coextrusion material for incorporating them into tissue simulants. First, the coextrusion material is loaded in its liquid state into a dispensing barrel. Next, the capsules are loaded into the barrel after the coextrusion material has undergone thermal gelation. Then, the dispensing barrel is placed in a planetary centrifugal mixer to mix the capsules into the coextrusion material to prepare a suspension that can be 3D printed. (**F**) The rheology of the coextrusion material-capsule suspension at various loadings. A volume fraction of 20% was chosen as acceptable for extrusion 3D printing. (**G**) Image of the 3D printed cric-skin tissue simulant with embedded capsules to simulate bleeding. (**H**) An image of the cric-skin puck after an incision demonstrating bleeding.

W-O-W DE capsules are typically formulated by trapping an aqueous inner phase (W_1_) within an organic middle phase (O) that is then suspended in a secondary aqueous outer phase (W_2_) (*56*). An ideal shell material for this application would need to be highly stable, i.e., allow minimal passive core fluid release, as well as provide facile rupturing on demand to release its contents. Polystyrene (PS) was selected as the shell material because of its robustness and resistance to degradation, with 15% w/w PS dissolved in dichloromethane forming the middle-phase solution. To mimic the viscosity and color of blood, 25% w/w glycerol in water-based red food coloring solution and 1% w/v polyvinyl alcohol (PVA) surfactant was used as the core solution. The glycerol weight fraction in this formulation is lower compared to that in standard blood-mimicking solutions (*14*) due to viscous contributions of red dye solution additives and PVA. To form the DEs, we used a custom capillary coflow microfluidic chip with three inlets and one outlet (fig. S10 and movie S3). Following the formation of the DE capsules (Fig. 4A), the shell solvent was removed using a rotary evaporator to form stable liquid core-polymer shell capsules (Fig. 4B). The capsules were stored in an osmotically balanced collecting solution, which ensured long-term stability without leakage (Fig. 4C).

The dimensions as well as the peak rupture force of the capsules could be tailored by adjusting the ratio of flow rates of the inner phase and the middle phase (fig. S11). An increase in the middle-phase flow rate relative to the inner-phase flow rate resulted in smaller capsules with thicker shells that were more difficult to rupture (Fig. 4D). Capsules manufactured with a 1:2 flow ratio between the inner and middle phases were chosen for further analysis as they provided a balance between capsule size and rupture force while being highly stable and monodisperse (fig. S12). As it is necessary to embed a large number of capsules within the tissue simulant to ensure sufficient volume of simulated blood, the capsules were mixed into a sacrificial fluidizing medium (Pluronic/glycerol hydrogel) to enable controlled dispensing (3D printing). To prepare a capsule-gel suspension, the carrier hydrogel was loaded into a syringe barrel in its liquid phase and then solidified via ambient warming. Then, capsules were added on top of the solid gel and mixed using a planetary centrifugal mixer (Fig. 4E). We studied the rheology of Pluronic/glycerol hydrogels at different volumetric capsule loadings to determine the ideal formulation for extrusion (Fig. 4F). Increasing the number of capsules in the suspension precipitously increased the shear storage modulus, likely due to the high stiffness of the PS shells. At 30% v/v capsule loading, the storage modulus was ~101 kPa, which is materially higher than the silicone-based inks developed above. Therefore, we chose 20% v/v (storage modulus of ~54 kPa) as the formulation of choice for embedding capsules into the tissue simulant.

To incorporate the blood-mimicking capsules within the tissue simulant, the skin layers of the simulant were 3D printed with silicone-based materials as previously described. Then, the capsule-gel suspension was extruded on top of the skin layer using a 10-GA tapered nozzle at an extrusion speed and pressure of 5 mm/s and 17.5 kPa, respectively (movie S4). The extruded capsule-gel suspension was then sealed by 3D printing the subcutaneous tissue layers with silicone-based materials on top of the extruded capsule-gel suspension (Fig. 4G). The tissue simulant was kept in ambient conditions for 24 hours to enable complete silicone curing. After curing, the tissue simulant was placed in a refrigerator to lower the temperature below its gel transition temperature (*T*_c_). This led to the liquefaction and removal of residual Pluronic/glycerol hydrogel, leaving the capsules embedded between the skin and the subcutaneous tissue layers of the tissue simulant. The capsules were then manipulated to release their contents by applying compression to crush the capsule shells. Last, an incision was made in the skin layer of the tissue simulant, opening the inner layers and allowing for leakage of blood-mimicking liquid to simulate bleeding (Fig. 4H and movie S5).

### Acceptability of the 3D printed cric-skin puck compared to cast cric-skin puck

To assess the impact of incorporating anisotropy in cricothyrotomy tissue simulants, we performed a comparative acceptability study in which participants performed successive cricothyrotomies on an isotropic cast cric-skin puck and the 3D printed anisotropic cric-skin puck. Active paramedics from the King County Medic One organization in Seattle, Washington, were recruited to perform an emergency cricothyrotomy on a female airway simulator (AJAMS). To avoid bias, the production of the cast cric-skin pucks was outsourced to an independent manufacturer. The 3D printed cric-skin pucks were manufactured with an anisotropic ratio of 1.5:1 and uniform directionality. Additionally, the 3D printed cric-skin puck was injected with an identical volume of simulated blood as the cast cric-skin puck to ensure equivalence. The participants watched a short refresher video prior to performing the cricothyrotomies (movie S6). Immediately after simulation, the participants completed a survey to assess the acceptability of the pucks. We hypothesized that the 3D printed puck would be at least equivalent to the cast puck.

The survey instrument consisted of 11 questions evaluating the two cric-skin pucks visually, physically, and behaviorally. The complete questionnaire and survey results can be found in table S1. The questions were scored on a scale from “not at all like human” (0) to “identical to human” (100). Thirteen participants were surveyed, with all but one completing the entire survey (one participant did not complete question 11).

Overall, this assessment found that 3D printed cric puck was more ideal for training cricothyrotomy than the cast puck (*P* = 0.029). There were no visual differences reported between the cast and 3D printed cric-skin (*P* = 0.074), subcutaneous tissue (*P* = 0.196), or cricothyroid membrane (*P* = 0.088) as compared to real human tissue. There were no physical differences reported between the cast and 3D printed cric puck’s simulated subcutaneous tissue as compared to a human neck while palpating (*P* = 0.100). When palpating, the simulated skin of the 3D printed puck most closely feels like the skin on a real human neck compared to the cast puck (*P* = 0.019). This was most likely due to the anisotropic mechanical behavior exhibited by 3D printed pucks.

Although there were no behavioral differences between the ability of the cast and 3D printed pucks to pinch like the skin of a real human neck (*P* = 0.365) or slide like the skin of a real human’s neck (*P* = 0.115), the 3D printed puck more closely replicated a human neck when cutting the simulated skin (*P* = 0.037), demonstrated more realistic bleeding (*P* = 0.008), and more accurately replicated the difficulty in recovering a lost incision site when placing an endotracheal tube through the cricothyroid membrane (*P* = 0.045).

## DISCUSSION

Certain high-acuity medical interventions are difficult to learn due to their low incidence in the general population. Tissue simulation is a necessity to ensure medical professionals improve their skills in performing such procedures. Current commercially available models are primarily made of silicone-based materials via molding or casting processes. The role of 3D printing thus far has been limited to creating molds for the casting process. In this research, we implemented a generalizable methodology to directly 3D print high-fidelity soft tissue simulants that can replicate the directional mechanics naturally observed in human tissue. Our core innovation exploits the inherent directionality of 3D printing and uses a simple overlapping line model to obtain the desired mechanical response while maintaining low printing time and high model quality. This is accomplished without needing to resort to using multiple materials to achieve anisotropy, although using multiple materials can further increase the anisotropic ratio. Multiple silicone-based inks can be used to manufacture simulants with a wide range of mechanical anisotropies while infill directions can be modified to closely mimic the natural alignment of collagen fibers within human tissue. A preliminary acceptability study indicated that 3D printed anisotropic cric-skin pucks performed better relative to cast pucks, with mechanical anisotropy being a key component of the improved performance. This result will need to be further verified with a larger-scale study to establish benchmarks for future simulation research. This research focuses on evaluating the impact of incorporating anisotropic mechanics into tissue simulants. The anisotropic nature of human tissue leads to a direction-dependent response to stretching and cutting operations, and replicating this property in tissue simulants results in improving their mechanical fidelity. Additional mechanical properties such as tear resistance, ultimate tensile strength, and elongation at break are important considerations for other simulation scenarios. The development of materials that mimic these mechanical properties can further improve simulation quality.

This work also demonstrates the direct 3D printing of relatively large DE capsules (~680 μm in diameter) uniformly embedded in a sacrificial shear-thinning matrix using a standard extrusion-based system. The temporary sequestration of fluid payloads from the surrounding tissue layers using DE capsules enables interference-free curing of silicones while preventing dehydration of the fluid payload until the simulation is performed. Next-generation tissue simulants can, therefore, potentially be manufactured in a single process. This may aid in rapidly scaling up the production quantity of these simulants, although the process remains inherently serial. This approach enables high-throughput deposition of liquid payloads in tissue simulants and may extend to broader applications requiring localized delivery of larger liquid volumes.

A limitation of our approach is that the predictiveness of the mathematical model is highly predicated on consistent deposition, which can be affected by air bubbles, incomplete mixing, and inaccurate initialization of home position. Another limitation is that the accuracy of deposition on inclined surfaces is lowered at higher angles of inclination (>45°). 3D printing platforms with additional DOFs can be developed to manufacture more intricate geometries of tissue simulants. The voxel parameters can also be adjusted throughout a printing process to yield complex gradient behaviors. Noncontact methods for enabling the release of capsule contents such as using ultrasonic waves can also be explored for ensuring breakage of all embedded capsules.

Advancing our anisotropic 3D printing workflow enables more realistic physiological test structures, enhancing medical devices and driving progress toward high-fidelity bionic organs. Future studies will focus on the following: (i) 3D printing tubular, spherical, and irregular-shaped tissue simulants; (ii) using materials such as hydrogels that can be tuned to the properties of human tissues and have properties that allow for the use of electrocautery, which is common during interventional procedures; (iii) automating capsule loading and delivery via the development of a thermo-electric mixing printhead; (iv) improving the drawability of filaments to reduce the susceptibility of print failure due to air bubbles; (v) incorporating vision systems to identify and automatically correct deposition errors such as under-extrusion; and (vi) modifying the underlying voxel design to incorporate nonlinear strain-stiffening mechanics of human tissue.

## MATERIALS AND METHODS

### Formulation and preparation of 3D printing material with tunable elastic modulus

The primary constituents of the 3D printing ink formulation were RTVS (LOCTITE SI-595 CL, Henkel Corp., Stamford, CT, USA) and PD (PD LV, Polytek Development Corp., Easton, PA, USA). FS (Fiberlay Inc., Sarasota, FL, USA) and FA (Bostick & Sullivan Inc., Santa Fe, NM, USA) were used as additives to improve the thixotropic and antistatic properties of the inks, respectively. Coloring pigments (FuseFX S-304, Daley Kreations, Ottawa, ON, Canada; and Functional Intrinsic II – Silicone Coloring System, Factor II Inc., Lakeside, AZ, USA) were added to match the color of the 3D printed tissue simulant to the advanced airway management manikins (AJAMS, UW CREST Lab, Seattle, WA, USA). The constituent materials were mixed at specific weight ratios to obtain inks of various elastic moduli and other mechanical properties. A planetary centrifugal mixer (ARE-310, Thinky U.S.A. Inc., Laguna Hills, CA, USA) was operated at 2000 rpm for 2 min and 2200 rpm for 2 min for mixing and defoaming the various ink formulations. The inks were then loaded on a 30-cc syringe barrel (Nordson EFD, Westlake, OH, USA), sealed with an end cap and a piston, and centrifuged at 2000 rpm for 2 min (Centrifuge 5430, Eppendorf North America, Enfield, CT, USA) to remove air bubbles. To separate the skin and subcutaneous tissue regions, a sacrificial ink consisting of 20% w/w Pluronic F-127 and 20% w/w glycerol in deionized (DI) water solution was used.

### Mechanical characterization of custom silicone-based materials

To characterize its softening effect, PD was mixed into RTVS at weight ratios of 0.00, 0.25, 0.50, 0.75, 1.00, 1.25, 1.50, 1.75, and 2.00. Constant quantities of FS, FA, and coloring pigments were also added to each of these inks. Each formulation was 3D printed into rectangular slabs (40 mm by 10 mm by 2 mm) with full infill and cured for at least 24 hours. These were subjected to uniaxial tensile testing at a strain rate of 1 mm/s and a maximum strain of 40% using a mechanical analyzer (RSA-G2, TA Instruments, New Castle, DE, USA). The elastic modulus of each sample was computed by fitting a fourth-degree polynomial (MATLAB polyfit) to the stress-strain curve and finding the tangent at 5% strain. Further, FS at weight fractions of 1, 2, 3, and 4% were mixed into a 1:1 formulation of RTVS and PD by weight to characterize the effect of FS on the elastic modulus of the mixture. The same printing and testing protocols described above were used to 3D print samples and measure the elastic modulus. The formulations that resulted in the closest match to the elastic moduli of skin and subcutaneous tissue were chosen for further process development.

### Print-line spanning characterization

To characterize the narrowing of printed lines when constructing the cellular voxel structure, a set of parallel lines with varying spacings was 3D printed on a flat glass slide using RTVS. Each test ink was subsequently loaded onto the volumetric dispensing printhead (vipro-HEAD 3, ViscoTec America Inc., Kennesaw, GA, USA) and printed at various print-line heights in the orthogonal direction. The extrusion flow rate was controlled such that the expected linewidth was equal to the nozzle diameter. The printed structure was fully cured for 24 hours under ambient conditions. For each test ink, the width of the spanning segments was measured using a microscope (DM4000 M, Leica Microsystems Inc., Deerfield, IL, USA) (*n* = 5). A second-degree polynomial was fitted to the measurements (MATLAB fit) to characterize the spanning behaviors for each ink.

### Rheological characterization of custom silicone-based inks

Rheological characterization of custom silicone-based inks was performed on a hybrid rheometer (Discovery HR20, TA Instruments) using a 40-mm stainless steel plate and a 2° cone and plate geometry at 22°C and a truncation distance of 50 μm. Storage and loss moduli of each ink were measured by conducting a logarithmic sweep of oscillatory shear stress from 1 to 1000 Pa at a frequency of 1 Hz. The complex viscosity of each ink was measured by conducting an oscillatory sweep up to 1% strain at frequencies ranging from 0.1 to 100 rad/s. These measurements were used as an analog to estimate the steady shear viscosity in accordance with the Cox-Merz rule.

### Deposition on inclined planes

Glass slides were mounted on an adjustable inclined-plane platform (AP180/M, Thorlabs Inc., Newton, NJ, USA) to serve as a test platform for studying deposition characteristics on inclined planes. Lines were deposited along the inclined planes under various nozzle offset conditions before being sprayed with a uniform layer of laser scanning aerosol (AESUB white, Scanningspray Vertriebs GmbH, Dortmund, Germany). The profiles of the deposited lines were then scanned using a laser displacement sensor (LK-H082, Keyence Corporation of America, Itasca, IL, USA) at a lateral resolution of 50 μm. The scanned data were then analyzed using MATLAB to identify mean deposition profiles for each case and validate ideal nozzle offset parameters.

### Manufacturing and 3D scanning of cric-puck substrate

A reusable deposition substrate in the shape of the bottom layer of the cric-skin puck was 3D printed using a Formlabs Rigid 4000 resin on a STL-based 3D printer (Form 2, Formlabs Inc., Somerville, MA, USA). To account for discrepancies between the digital model and the 3D printed substrate, a laser displacement sensor (LK-H082, Keyence Corporation of America) was used to scan the substrate area at a resolution of 100 μm. The raw scan data were imported into MATLAB and converted into a smooth, uniform mesh using linear interpolation.

### 3D printing of cric-skin puck

The substrate was mounted on a stationary platform and aligned to the *x* and *y* axes of our custom 3D printing gantry (AGS1000, Aerotech Inc., Pittsburgh, PA, USA). A silicone mold release spray (Ease Release 200, Mann Release Technologies Inc., Macungie, PA, USA) was used to apply a coating on the substrate for easy removal postprinting. A volumetric dispensing printhead (vipro-HEAD 3, ViscoTec America Inc.) was mounted on the *z* axis of the gantry and was used to ensure viscosity-independent deposition of materials. A digital pneumatic regulator (Ultimus V, Nordson EFD) was used to establish a continuous material feed into the printhead reservoir, and the printhead was driven using a microcontroller (Arduino Uno). The inks for the skin and subcutaneous tissue sections were sequentially loaded onto the printhead, and each section was printed with specific infill print-line heights and print-line spacings depending on the desired anisotropy. A general-purpose 18-GA dispensing tip (GP 0.033 × 0.25, Nordson EFD) was used to deposit both inks. Cover layers of thickness equal to 600 μm were printed with full infill to ensure that the external surfaces of the cric pucks were smooth. The printing speed was maintained at a constant 16.667 mm/s, and the extrusion rate was controlled on the basis of the layer height such that the extruded filament had the same width as the nozzle inner diameter. The skin and subcutaneous tissue sections were separated by printing a thin layer of a Pluronic-based hydrogel as a sacrificial support layer. This allowed trainees to pinch and slide the skin layer over the subcutaneous tissue layer to identify the location of the cricothyroid membrane. After printing, the models were left to cure in ambient air for at least 24 hours to ensure full curing. The sacrificial ink was removed via reverse gelation by placing the cured 3D printed tissue simulant in a 4°C refrigerator.

### Mechanical characterization of cric-skin puck

The mechanical anisotropy of the 3D printed cric-skin puck was measured by extracting square (30 mm by 30 mm) samples from various locations within the puck. These samples were further cut into a cruciform shape by stamping out 10 mm–by–10 mm squares from each of the corners. The cruciform samples were mounted on the mechanical analyzer (RSA-G2, TA Instruments) and sequentially subjected to uniaxial tensile testing along the two orthogonal axes using the same protocol described above. The elastic modulus along each axis was computed by fitting a fourth-order polynomial (MATLAB polyfit) to the stress-strain curve and finding the tangent at 5% strain. The mechanical anisotropy was then measured by computing the ratio between the elastic moduli along the two orthogonal axes.

### Fabrication of the microfluidic chip for fluid-filled capsule development

Before microfluidic chip fabrication, square capillary tubes (VitroTubes, VitroCom, Mountain Lakes, NJ, USA) with an inner edge length of 0.8 mm, an outer edge length of 1.12 mm, and a length of 50 mm were treated with piranha solution (3:1 ratio of sulfuric acid and 30% hydrogen peroxide). This chemical treatment removes organic residue from the inner walls of the glass tubing, thereby reducing potential hydrophobic interactions with the DE oil phase and providing stable capsule output. Microfluidic chips for producing DEs were developed by first cutting an ~6- to 8-cm-length piece of polyvinyl chloride (PVC) tubing (McMaster-Carr Supply Company, Elmhurst, IL, USA) and a 2- to 3-cm piece of 30 AWG Teflon tubing (McMaster-Carr). Next, a 32-GA nozzle (GP 0.004 × 0.25, Nordson EFD) was inserted through the outer wall of the PVC tubing (~1 cm away from one end of the tube) and pushed through the inner lumen and out through the end of the PVC tubing. The Teflon tubing was then inserted as a sheath covering the 32-GA nozzle and pushed through the space between the 32-GA needle and the PVC tubing lumen. This connection, which facilitates the water-in-oil emulsification of the inner phase (core) in the middle phase (shell), was then sealed together with epoxy to prevent leakage.

A 6- to 8-cm-length piece of polyethylene tubing (BD Intramedic, BD, Franklin Lakes, NJ, USA) was then cut, and the opening at one of the tube ends was mildly stretched using tweezers to facilitate a tight fit of the glass capillary tubing inside the lumen. The glass capillary tubing was inserted ~1 to 1.5 cm inside the polyethylene tubing. Next, the tip of a 20-GA nozzle (GP 0.024 × 1, Nordson EFD) was heated by a soldering iron and used to puncture a hole in the wall of the polyethylene tubing where the glass capillary tubing insert ends. The glass capillary tubing was then removed, and the Teflon end of the 32-GA–Teflon–PVC epoxied construct was inserted in the 20-GA hole and pushed through the polyethylene tubing opening. The Teflon sticking out of the end of the polyethylene tubing was cut to only extend <0.5 cm. The glass capillary tubing was then carefully reinserted into the polyethylene tubing, with the extended Teflon fitting inside the capillary tubing, while the glass capillary tubing fit tightly inside the polyethylene tubing. Through this design, the single emulsions created through the Teflon tubing were emulsified again in the hydrophilic outer phase that flows through the polyethylene tubing, creating W-O-W DEs, which travel out of the chip through the connected glass capillary tubing. The connection of the 32-GA–Teflon–PVC construct to the polyethylene tubing as well as the connections between the capillary tubing, Teflon, and polyethylene tubing were all epoxied together to prevent leakage. The microfluidic chip was left in ambient conditions for at least 90 min after the second application of epoxy for more robust curing.

### Formulation and preparation of fluid-filled capsules

For fabrication of DE blood-mimicking capsules, a capsule core solution (inner phase) containing 25% w/w glycerol (Sigma-Aldrich, St. Louis, MO, USA), 75% w/w water-based red food coloring solution (McCormick, Cockeysville, MD, USA), and 1% w/v PVA (MW 31,000-50,000, 87 to 89% hydrolyzed, Sigma-Aldrich); a capsule shell (middle phase) solution containing 15% w/v PS (MW 45,000, Scientific Polymer Products Inc., Ontario, NY, USA) in dichloromethane (Honeywell Burdick & Jackson, Muskegon, MI, USA); and a flow (outer phase) solution containing 25% w/w glycerol and 1% w/v PVA in DI water were prepared. Each solution was loaded onto a syringe pump and connected to a custom-developed microfluidic chip, where the inner-phase syringe was connected to the 32-GA nozzle on the chip, the middle phase was connected to PVC tubing on the chip, and the outer phase was connected to polyethylene tubing on the chip.

The syringe pump-controlled flow rates for the inner, middle, and outer phases were 0.05, 0.1, and 0.5 ml/min, respectively. To begin fabrication, the outer-phase flow was first turned on, and, then, the middle-phase flow was additionally turned on to create initial oil-in-water single emulsions. Next, the inner-phase flow was turned on to emulsify the core solution in the shell previously emulsified in the outer flow, thus creating W-O-W DEs. DEs flow out of the microfluidic chip through the square glass capillary tube, and the capsules were collected in the same solution composing the outer phase (collecting solution). Next, a glass vial or bottle containing the blood-mimicking capsules in collecting solution was connected to a rotary evaporator system (RV 10, IKA Works Inc., Wilmington, NC, USA). Dichloromethane in the oil phase of the DEs was evaporated under 600 mbar of vacuum pressure and 45-rpm rotation overnight. After shell solvent evaporation, the capsules were stored long-term in a collecting solution with glycerol content matching the capsule core (25% w/w glycerol in DI water) to maintain osmotic stability and prevent core dye leakage.

### Rheological characterization of fluid-filled capsules

The dynamic viscosity of different capsule fluid core formulations (15, 20, 25, 30, 35, and 40% w/w glycerol in red food dye solution and 1% w/v PVA) was assessed with the same hybrid rheometer previously described (HR-20, TA Instruments), but instead using a stainless steel AR-G2 double wall concentric cylinder geometry with an outside bob diameter of 35 mm and an outside cup diameter of 37 mm. Samples were subjected to a shear rate flow sweep of 1 to 100 s^−1^ at 23°C. The dynamic viscosity used for formulation assessment was the value measured at the max shear rate, where minimal shear thinning was observed for all samples.

Using the same rheometer, the rheological characterization of capsules suspended in Pluronic was performed using a sandblasted 20-mm stainless steel parallel plate geometry at 22°C. Capsules were loaded into a mixture of 20% w/w Pluronic F-127 and 20% w/w glycerol hydrogel at 10, 20, and 30% loadings by volume (v/v). Storage and loss moduli of each suspension were measured by conducting a logarithmic sweep of oscillatory shear stress from 1 to 1000 Pa at a frequency of 1 Hz.

### Compression testing and sizing of fluid-filled capsules

Compression tests were performed on the fluid-filled capsules using a mechanical analyzer (RSA-G2, TA Instruments) with 8-mm flat discs and a constant linear rate of 5 μm/s. Three sets of capsules were tested: (i) capsules fabricated using a 1:3 inner:middle flow ratio (0.05 ml/min:0.15 ml/min), (ii) a 1:2 inner:middle flow ratio (0.05 ml/min:0.1 ml/min), and (iii) a 1:1 inner:middle flow ratio (0.1 ml/min:0.1 ml/min). For the 1:1 flow ratio capsules, a core solution of 15% w/w glycerol in red food coloring solution was used instead of 25% w/w glycerol in red food coloring solution to increase stability after fabrication. Compression force was exerted on the samples past the point of complete failure (i.e., capsule breakage). The diameter of each capsule before compression was determined by finding the gap width between the plates at which the first marked increase in compression force was registered by the transducer. The capsule size distribution for the 1:2 flow ratio condition (i.e., the formulation used for incorporation into the 3D printed tissue simulants) was also measured using a laser diffraction particle size analyzer (Microtrac Inc., Montgomeryville, PA, USA).

### Stability of fluid-filled capsules

To measure the release of red dye from the fluid core, 140 capsules were placed in a vial of ~2 ml of collecting solution (25% w/w glycerol and 1% w/v PVA) and stored at room temperature (*n* = 3). The collecting solution was sampled and transferred to 96-well plates every 5 days for 40 days and measured for absorbance using a plate reader (BioTek Cytation 5, Agilent, Santa Clara, CA, USA). The percentage release of red dye for each timepoint was calculated by comparing the absorbance to samples containing 140 capsules completely ruptured via compression in 2 ml of collecting solution along a preset calibration curve. The absorbance peak of the red food coloring solution (530 nm) was determined by conducting a sweep between 300 and 700 nm with the plate reader. Stability, shape retention, and color retention of the capsules were also tested via observation on a glass slide in a dry state over a period of 2 days (*n* = 3).

### Preparation of fluid-filled capsule ink for 3D printing

To prepare a capsule-based ink for 3D printing, a 10-cc syringe barrel (Nordson EFD) was injected with RTVS to plug the narrow Luer-lock connection region at the cartridge bottom. The barrels were left overnight at room temperature to allow for the silicone to fully cure. After silicone curing, blood mimicking capsules that had undergone evaporation were filtered by size using a 500-μm-sized metal mesh to remove PS particles which failed to maintain a DE liquid core. The DEs were then transferred into DI water and counted. A mixture of 20% w/w Pluronic F-127 and 20% w/w glycerol in DI water was cooled below its *T*_c_ (~10°C) and pipetted into the syringe barrel, where it rapidly rewarmed and solidified. The capsules were then transferred in water via a scored glass pipet on top of the solidified gel at a 20% v/v ratio. The volume of each capsule was calculated using the equation below

$$v_{c}=\frac{4\pi {r_{c}}^{3}}{3}$$ (6)

where *r*_c_ = 337.5 μm (based on an approximate average capsule diameter of 675 μm). Therefore, the volume of each capsule was estimated to be 0.161 μl, and the total added volume of capsules as well as the corresponding volume of added gel medium was determined from this estimate.

To distribute capsules within the gel medium, water transferred during the capsule loading process was removed, and the contents in the syringe barrel were mixed at 2000 rpm for 2 min in a planetary centrifugal mixer (ARE-310, Thinky U.S.A. Inc.). Next, a flat wall piston (Nordson EFD) was inserted down into the syringe barrel until it was in contact with the capsule-loaded ink. Last, the silicone plug was carefully removed from the bottom of the syringe barrel and a 10-GA tapered nozzle (Fisnar QuantX, Germantown, WI, USA) was attached before being connected to the Aerotech printing system for dispensing.

### Statistical analysis

The study was conducted with an exemption approval from the University of Washington Institutional Review Board (STUDY00018210). Data were analyzed via a series of paired-sample *t* tests. As the paired-sample *t* test assumes an approximately normal distribution of data across the two levels of the independent variable, normality was assessed via the Shapiro-Wilk test of normality and a normal Q-Q plot. Additionally, as the paired-sample *t* test assumes that there are no significant outliers in the differences between the related groups, box plots were created to visually detect the presence of outliers in the data. For these purposes, outliers were defined as data points in excess of 1.5 box lengths from the edge of the box in the boxplot. In instances where either of these two assumptions was violated (outliers were detected, or data were not normally distributed), the Wilcoxon signed-rank test was adopted as a nonparametric alternative to the paired-sample *t* test (e.g., for questions 4, 7, 9, and 10). Data interpreted parametrically were reported as means and SDs, while data interpreted nonparametrically were reported as medians and interquartile ranges. In either case, statistical significance was defined as *P* < 0.05. All analyses were conducted via SPSS Statistics (version 28, IBM Corp., Armonk, NY, USA).

## Supplementary Materials

### The PDF file includes:

Supplementary Text Figs. S1 to S12 Table S1 Legends for movies S1 to S6 References
