# Supplementary material for: 3D printed anisotropic tissue simulants with embedded fluid capsules for medical simulation and training
Source: Sci Adv. 2025 Aug 29;11(35):eadw6446. doi: 10.1126/sciadv.adw6446 (PMC12396343; doi:10.1126/sciadv.adw6446)
Supplement: Supplementary file 1 — Supplementary Text Figs. S1 to S12 Table S1 Legends for movies S1 to S6 References [file sciadv.adw6446_sm.pdf]

Science Advances
AAAS

Supplementary Materials for

3D printed anisotropic tissue simulants with embedded fluid capsules for medical simulation and training

Adarsh Somayaji *et al.*

Corresponding author: Michael C. McAlpine, mcalpine@umn.edu

Sci. Adv. **11**, eadw6446 (2025)
DOI: 10.1126/sciadv.adw6446

The PDF file includes:

Supplementary Text
Figs. S1 to S12
Table S1
Legends for movies S1 to S6
References

Other Supplementary Material for this manuscript includes the following:

Movies S1 to S6

Supplementary Text

Supplement 1: Mathematical model for anisotropy

Fundamental Solid Body Equations:

$$\sigma = \varepsilon * E \quad (1)$$

$$\frac{F}{A} = \frac{\Delta L}{L} * E \quad (2)$$

Volume Relations used for simplification:

$V_{L1} = w_1 h_1 L_1; V_{L2} = w_2 h_2 L_2; V_{S1} = L_1 h_1 (L_2 - w_1); V_{S2} = L_2 h_2 (L_1 - w_2); V_B = L_1 L_2 (h_1 + h_2)$

Longitudinal Deformation:

From Fig. S1A and (1) and (2)

$$\frac{F_L}{L_2 * (h_1 + h_2)} = \left(\frac{\Delta L_1}{L_1}\right) E_L \quad (3)$$

The change in length ΔL_1 can be decomposed as follows:

$$\Delta L_1 = \Delta S_L + \Delta B_L \quad (4)$$

$$\left(\frac{\Delta S_L}{L_1 - w_2}\right) E_1 = \frac{F_L}{h_1 * w_1} \quad (5)$$

$$\left(\frac{\Delta B_L}{w_2}\right) E_{B_L} = \frac{F_L}{(h_1 + h_2) * L_2} \quad (6)$$

Substituting (5) and (6) in (4), we get

$$\Delta L_1 = \frac{F_L * (L_1 - w_2)}{(E_1 * h_1 * w_1)} + \frac{F_L * w_2}{(E_{B_L} * (h_1 + h_2) * L_2)} \quad (7)$$

Considering this subsystem B_L as springs in parallel (Fig. S1B),

$$F_L = F_{L1} + F_{L2} \quad (8)$$

Expanding (8) using (2), we get

$$\left(\frac{\Delta B_L}{w_2}\right) E_{B_L} * (h_1 + h_2) * L_2 = \frac{E_1 * \Delta B_L * h_1 * w_1}{w_2} + \frac{E_2 * \Delta B_L * h_2 * L_2}{w_2} \quad (9)$$

Simplifying (9), we get

$$E_{B_L} = \frac{E_1 * h_1 * w_1 + \ E_2 * h_2 * L_2}{(h_1 + h_2) * L_2} \quad (10)$$

Substituting (10) in (7)

$$\Delta L_1 = \frac{F_L * (L_1 - w_2)}{(E_1 * h_1 * w_1)} + \frac{F_L * w_2 * \ (h_1 + h_2) * L_2}{(E_1 * h_1 * w_1 + \ E_2 * h_2 * L_2 * (h_1 + \ h_2) * L_2)} \quad (11)$$

Substituting (11) in (3)

$$E_L = \frac{(E_1 * h_1 * w_1 * L_1) * (E_1 * h_1 * w_1 + E_2 * h_2 * L_2)}{L_2 * (h_1 + h_2) * (E_1 * h_1 * w_1 * L_1 + E_2 * h_2 * L_2 * (L_1 - w_2)} \quad (12)$$

Simplifying using volume relations, we get (13) as the elastic modulus along the longitudinal direction.

$$E_L = \frac{(V_{L1} * E_1 * (V_{L1} * E_1 + (V_{S2} + \ V_{L2}) * E_2))}{\left(V_B * (V_{L1} * E_1 + V_{S2} * E_2)\right)} \quad (13)$$

Transverse deformation:
From Fig. S1C and (1) and (2),

$$\frac{F_T}{L_1 * (h_1 + h_2)} = \left(\frac{\Delta L_2}{L_2}\right) E_T \quad (14)$$

The change in length δL_2 can be decomposed as follows:

$$\Delta L_2 = \ \Delta S_T + \Delta B_T \quad (15)$$

$$\left(\frac{\Delta S_T}{L_2 - w_1}\right) E_2 = \frac{F_T}{h_2 * w_2} \quad (16)$$

$$\left(\frac{\Delta B_T}{w_1}\right) E_{B_T} = \frac{F_T}{(h_1 + h_2) * L_1} \quad (17)$$

Substituting (16) and (17) in (15), we get

$$\Delta L_2 = \frac{F_T * (L_2 - w_1)}{(E_2 * h_2 * w_2)} + \frac{F_T * w_1}{(E_{B_T} * (h_1 + \ h_2) * L_1)} \quad (18)$$

Considering this subsystem B_T as springs in parallel (Fig. S1D),

$$F_T = \ F_{T1} + F_{T2} \quad (19)$$

Expanding (19) using (2), we get

$$\left(\frac{\Delta B_T}{w_1}\right) E_{B_T} * (h_1 + h_2) * L_1 = \frac{E_2 * \Delta B_T * h_2 * w_2}{w_1} + \frac{E_1 * \Delta B_T * h_1 * L_1}{w_1} \quad (20)$$

Simplifying (20), we get

$$E_{B_T} = \frac{E_2 * h_2 * w_2 + E_1 * h_1 * L_1}{(h_1 + h_2) * L_1} \quad (21)$$

Substituting (21) in (18), we get

$$\Delta L_2 = \frac{F_T * (L_2 - w_1)}{(E_2 * h_2 * w_2)} + \frac{F_T * w_1 * (h_1 + h_2) * L_1}{(E_2 * h_2 * w_2 + E_1 * h_1 * L_1 * (h_1 + h_2) * L_1)} \quad (22)$$

Substituting (22) in (14)

$$E_T = \frac{(E_2 * h_2 * w_2 * L_2) * (E_2 * h_2 * w_2 + E_1 * h_1 * L_1)}{L_1 * (h_1 + h_2) * (E_2 * h_2 * w_2 * L_2 + E_1 * h_1 * L_1 * (L_2 - w_1)} \quad (23)$$

Simplifying using volume relations, we get (24) as the elastic modulus along the transverse direction.

$$E_T = \frac{(V_{L2} * E_2 * (V_{L2} * E_2 + (V_{S1} + V_{L1}) * E_1))}{\left(V_B * (V_{L2} * E_2 + V_{S1} * E_1)\right)} \quad (24)$$

From (13) and (24), we can compute the mechanical anisotropy as a function of voxel parameters using (25)

$$\gamma = \frac{E_L}{E_T} \quad (25)$$

<u>Supplement 2: Nozzle height adjustments for deposition on non-planar substrates</u>

Consider the deposition case of printing up an inclined plane (Fig. S4A (left)).
The desired layer height h_0 = BE and CB = $\frac{d}{2}$
Draw a line CF parallel to BE.
Drop a perpendicular from C intersecting BE at G.

$$\angle BCG = \alpha$$

$$BG = CB * \sin\alpha = \left(\frac{d}{2}\right) * \sin\alpha$$

$$CE = BE - BG = h_0 - \left(\frac{d}{2}\right) * \sin\alpha$$

$$\angle FCD = \alpha$$

$$h = CD = \frac{CF}{\cos \alpha} = \frac{h_0}{\cos \alpha} - \left(\frac{d}{2}\right) * \tan \alpha \quad (26)$$

Consider the deposition case of printing down an inclined plane (Fig. S4B (right)).
The desired layer height h_0 = AE and AC = $\frac{d}{2}$
Draw a line CF parallel to AE.
Drop a perpendicular from A intersecting CF at G.

$$\angle CAG = \alpha$$

$$CG = AC * \sin \alpha = \left(\frac{d}{2}\right) * \sin \alpha$$

$$CF = GF + CG = h_0 + \left(\frac{d}{2}\right) * \sin \alpha$$

$$\angle FCD = \alpha$$

$$h = CD = \frac{CF}{\cos \alpha} = \frac{h_0}{\cos \alpha} + \left(\frac{d}{2}\right) * \tan \alpha \quad (27)$$

Equations (26) and (27) define the adjustments necessary to position the nozzle for accurate deposition on inclined surfaces.

<u>Supplement 3: Variation in anisotropy with change in orientation relative to measurement axes</u>

Consider the measurement axes oriented at an angle ϕ relative to the orthogonal print lines (Fig. S5A).
If a force F is applied along the X' axis, it can be resolved into its components along the X and Y axes as $F * \cos \phi$ and $F * \sin \phi$ respectively. Equation (3) can be applied as follows:

$$\frac{F * cos\ \phi}{L_2 * (h_1 + h_2)} = \left(\frac{\Delta L_{11}}{L_1}\right) E_L$$

If we constrain L_1 to be equal to L_2 (i.e. line spacings are equal for both layers),

$$\Delta L_{11} = \frac{F * cos\ \phi}{E_L * (h_1 + h_2)}$$

Similarly, the displacement along the Y axis is given by

$$\Delta L_{21} = \frac{F * sin\ \phi}{E_T * (h_1 + h_2)}$$

Therefore, the net displacement due to the applied force F is given by

$$\Delta L_1 = \sqrt{\Delta L_{11}{}^2 + \Delta L_{21}{}^2}$$

$$\Delta L_1 = \frac{F}{(h_1 + h_2)} \sqrt{\frac{cos^2(\phi) * E_T^2 + sin^2(\phi) * E_L^2}{E_L^2 * E_T^2}} \quad (28)$$

Now, consider the same force F applied in the orthogonal direction Y' (Fig. S5B). It can be resolved into its components along the X and Y axes as $F * \sin \phi$ and $F * \cos \phi$ respectively. Equation (3) can be applied as follows:

$$\frac{F * sin\ \phi}{L_2 * (h_1 + h_2)} = \left(\frac{\Delta L_{12}}{L_1}\right) E_L$$

If we constrain L_1 to be equal to L_2

$$\Delta L_{12} = \frac{F * sin\ \phi}{E_L * (h_1 + h_2)}$$

Similarly, the displacement along the Y axis is given by

$$\Delta L_{22} = \frac{F * cos\ \phi}{E_T * (h_1 + h_2)}$$

Therefore, the net displacement due to the applied force F is given by

$$\Delta L_2 = \sqrt{\Delta L_{12}{}^2 + \Delta L_{22}{}^2}$$

$$\Delta L_1 = \frac{F}{(h_1 + h_2)} \sqrt{\frac{sin^2(\phi) * E_T^2 + cos^2(\phi) * E_L^2}{E_L^2 * E_T^2}} \quad (29)$$

The mechanical anisotropy can be computed by finding the ratio of the displacements as follows:

$$\gamma = \frac{\Delta L_2}{\Delta L_1} \quad (30)$$

Substituting (28) and (29) in (30), we get

$$\gamma(\phi) = \frac{\sqrt{E_L^2 * cos^2(\phi) + E_T^2 * sin^2(\phi)}}{\sqrt{E_L^2 * sin^2(\phi) + E_T^2 * cos^2(\phi)}} \quad (31)$$

Supplement 4: PSOCT imaging system and measurements

The light source utilized in this study was a 200 kHz swept laser source (Axsun, Excelitas Technologies Corp., Pittsburgh, PA, USA), featuring a central wavelength of 1310 nm and a spectral tuning range of 100 nm. A coupler (90/10) split the laser output, directing it to the sample arm and reference arm. The sample arm incorporated a commercial handheld probe (OCTG 1300 NR, Thorlabs Inc.) and a calibration target for swift digital calibration and polarization mode dispersion (PMD) compensation. The calibration target, a ringlike polylactic acid 3D printer filament, had its fast optic axis aligned along the circle. Reflections from both sample and reference arms were recombined by a 50/50 coupler and directed to the polarization detection unit. Here, coherence signals were split into orthogonal polarization components using two polarization beam splitters and detected by photodetectors. The polarization from the reference arm underwent adjustments to ensure equal power in the two polarization channels via polarization controllers. Notably, the automatic digital calibration of the system eliminated the need for a specific known input polarization state. Exploiting this feature, an additional polarization controller in the sample arm was fine-tuned to generate an input polarization state optimizing sensitivity and avoiding measurements with low sensitivity.

The study employed a polarization state tracing method to extract depth-resolved phase retardation and axis orientation from PSOCT measurements. In this methodology, Stokes parameters represented the output polarization states. Digital PMD compensation was achieved by dividing the OCT spectrum into five bins with distinct central wavelengths (*57*). For each bin, depth-resolved local optics axis information was derived by averaging the Stokes parameters (using a 6×6 spatial averaging filter) and normalizing them to the surface of the Poincare sphere. The trajectory of the Stokes vectors in each A-scan at the Poincare sphere formed a spatial curve.

Discrete differential geometry was then applied to this curve to provide TNB vectors (T: tangent vector, N: normal vector, B: binormal vector of the curve) (*52*, *53*). The relation between the binormal vectors and the local optics axis, as well as local phase retardation of the sample, had been established in a previous study and was expressed as follows:

$$\delta_n = \frac{1}{2}\frac{N_{n-1}.N_n}{|N_{n-1}||N_n|} \quad (32)$$

$$A_n = R_{n-1}(-\delta_{n-1}; A_{n-1})R_{n-2}(-\delta_{n-2}; A_{n-2}) \ldots \ldots R_1(-\delta_1; A_1)B_n \quad (33)$$

where N_n, $B_n$are the normal and binomial vectors of the spatial curve, $R_n(-\delta_n; A_n)$ is the 3D rotation matrix determined by A_n and $-\delta_n$. By aligning the output obtained from each bin to the central bin, the PMD is compensated digitally without the need for an extra input polarization measurement. In this system, the PSOCT was performed with the objective lens (LSM03, Thorlabs Inc.), providing a lateral resolution of 25 μm. The axial resolution was approximately 7.5 μm in air. To enhance the signal-to-noise ratio, four repeated B-scans were conducted at each location, and the interference signals were averaged before processing. The complete volumetric scanning, comprising 500 A-lines in the Y-axis direction and 2000 A-lines in the X-axis direction, was completed in 4.2 seconds.

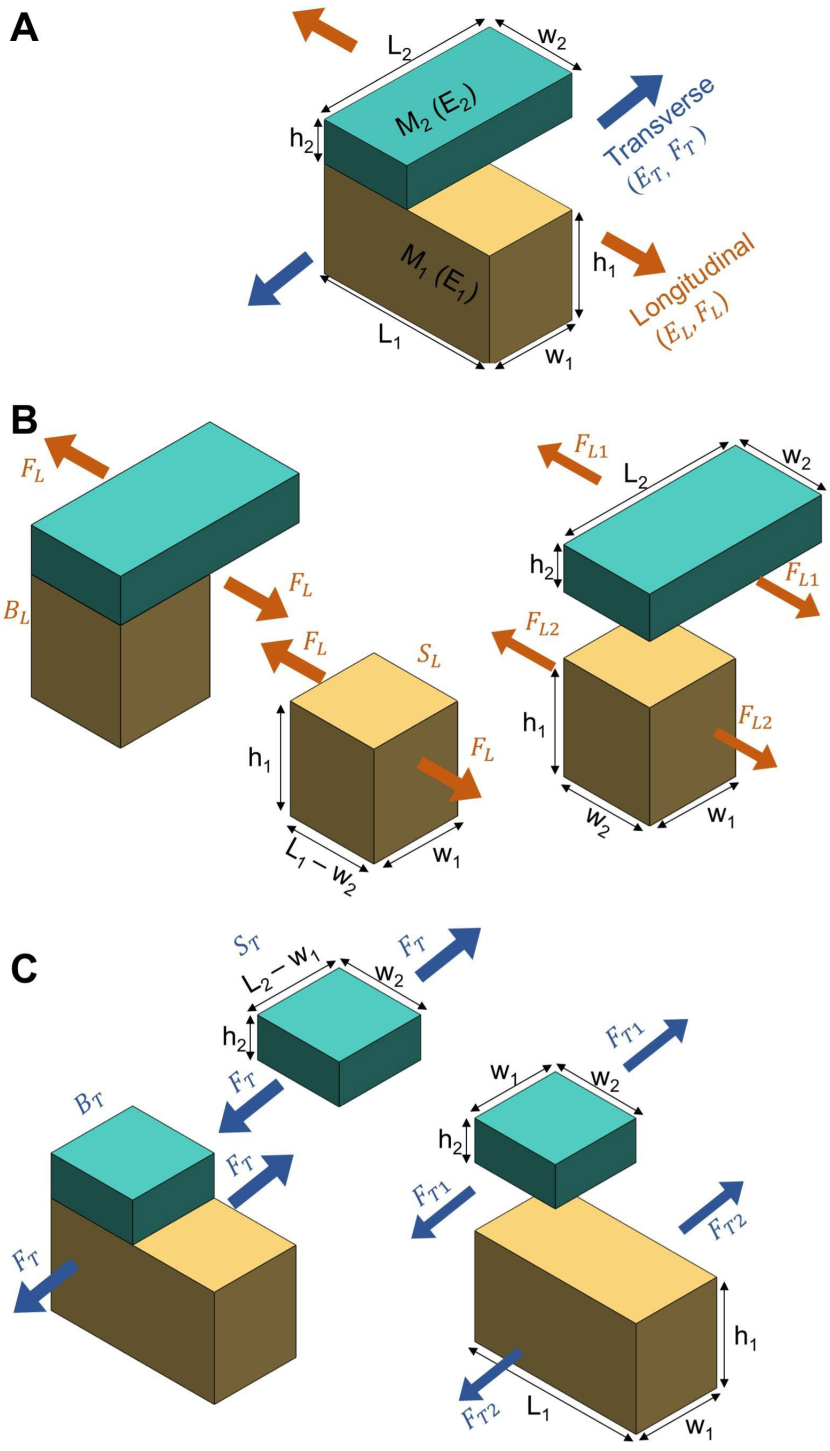

Figure S1. Voxel anisotropy model derivation. (**A**) Base voxel structure with print line variables and materials labelled. The mechanical anisotropy exhibited by the structure is given by the ratio of the stiffnesses in the longitudinal and transverse directions. (**B**) Decomposition of the voxel structure under applied forces in the longitudinal direction. (**C**) Decomposition of the voxel structure under applied forces in the transverse direction.

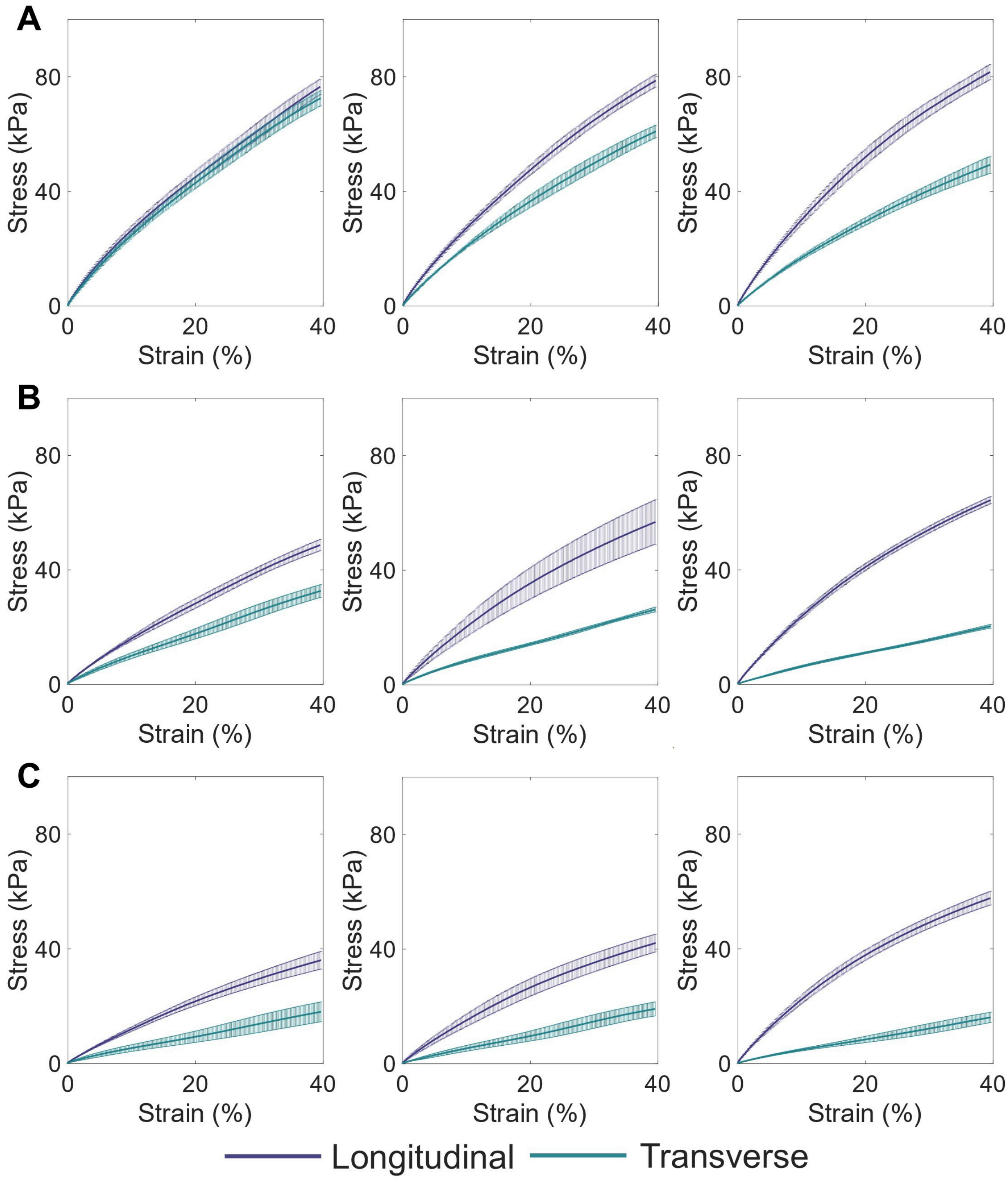

Figure S2. Tensile testing of multi-material anisotropic samples for each set of input materials. Height ratios are as follows: $h_1/h_2 = 1$ (left), $h_1/h_2 = 2$ (middle), and $h_1/h_2 = 3$ (right). (**A**) $E_1/E_2 = 1.0$ (**B**) $E_1/E_2 = 3.5$ (**C**) $E_1/E_2 = 5.5$ ($n = 3$, error bars indicate standard deviation)

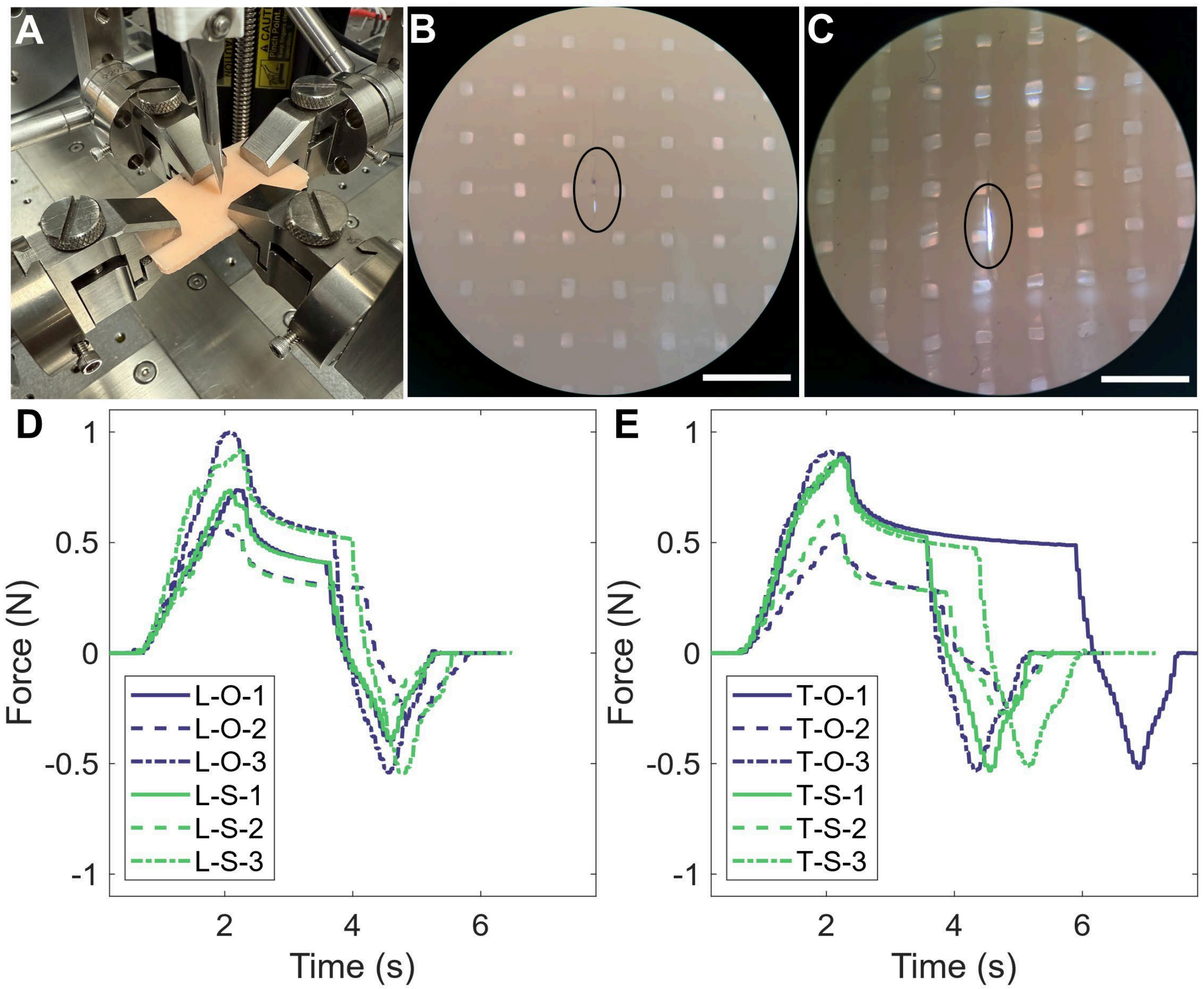

F

Test scenario	Peak puncture force (N)
Longitudinal direction and overlapping section	0.771 ± 0.212
Longitudinal direction and spanning section	0.749 ± 0.164
Transverse direction and overlapping section	0.780 ± 0.210
Transverse direction and spanning section	0.789 ± 0.148

Figure S3. Puncture force characterization of 3D printed anisotropic samples. (**A**) Experimental setup for performing puncture tests. (**B**) Microscope image of sample punctured on the overlapping section. The puncture location is indicated (Scale bar = 2 mm). (**C**) Microscope image of sample punctured on the spanning section. The puncture location is indicated (Scale bar = 2 mm). (**D**) Puncture force vs time plots for each test case in the longitudinal direction. (**E**) Puncture force vs time plots for each test case in the transverse direction. (Naming convention: L – longitudinal direction; T- transverse direction; O – overlapping section; S- spanning section). (**F**) Comparison of mean and standard deviation of peak puncture force between different test scenarios (n = 3).

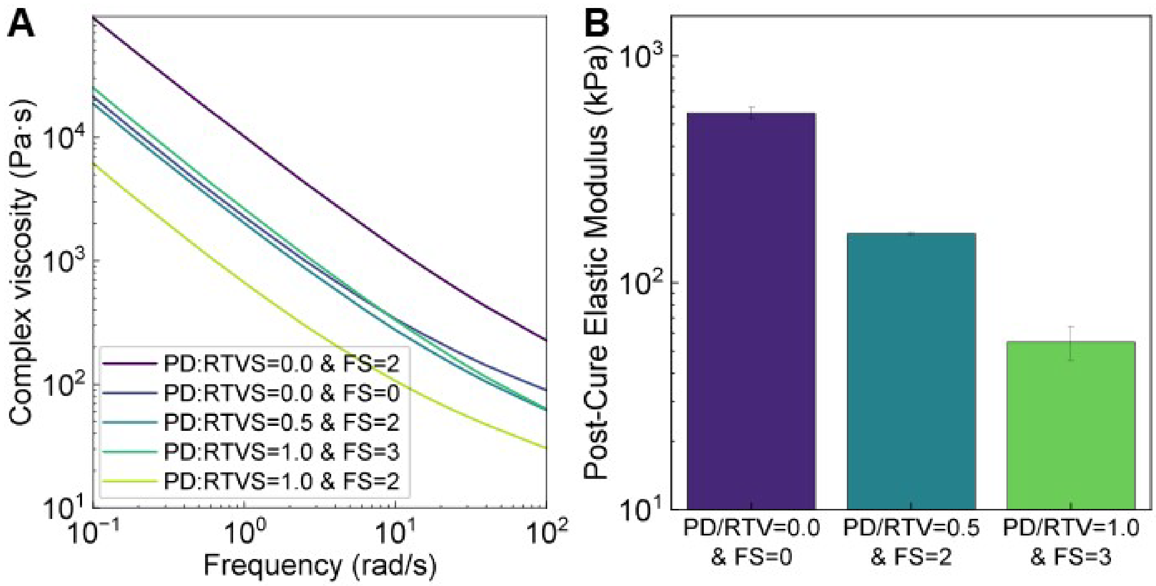

C

Formulation (in deionized water)	Avg. pH	Avg. Z.P. (mV)
0.1% w/w FS	6.2	-51.7
0.09% w/w FS + 0.01% w/w FA	6.1	-55.9
**0.08% w/w FS + 0.02% w/w FA**	**5.6**	**-0.1**
**0.07% w/w FS + 0.03% w/w FA**	**5.4**	**-0.0**
**0.06% w/w FS + 0.04% w/w FA**	**5.3**	**0.0**
0.05% w/w FS + 0.05% w/w FA	5.3	31.5
0.025% w/w FS + 0.075% w/w FA	5.2	28.7
0.1% w/w FA	5.2	30.8

Figure S4. Additional material characterization results. (**A**) Viscometry of silicone-based inks shows that by adding specific quantities of fumed silica relative to PlatSil Deadener and RTV silicone, the complex viscosity of inks can be tuned to have near-identical properties. Here, inks with PD:RTVS=0.0 & FS=0, PD:RTVS=0.5 & FS=2, and PD:RTVS=1.0 & FS=3 have almost identical shear-thinning properties. (**B**) The post-cure elastic moduli of the 3 inks with similar rheological behaviors are shown for comparison. (**C**) Variation in average Zeta potential (Z.P.) and pH at different weight ratios of fumed silica (FS) and fumed alumina (FA) in deionized water. The range of values for which the Z.P. is zero is highlighted in bold. FS: FA weight ratios ranging from 1.5:1 to 4:1 showed a stable net zero potential.

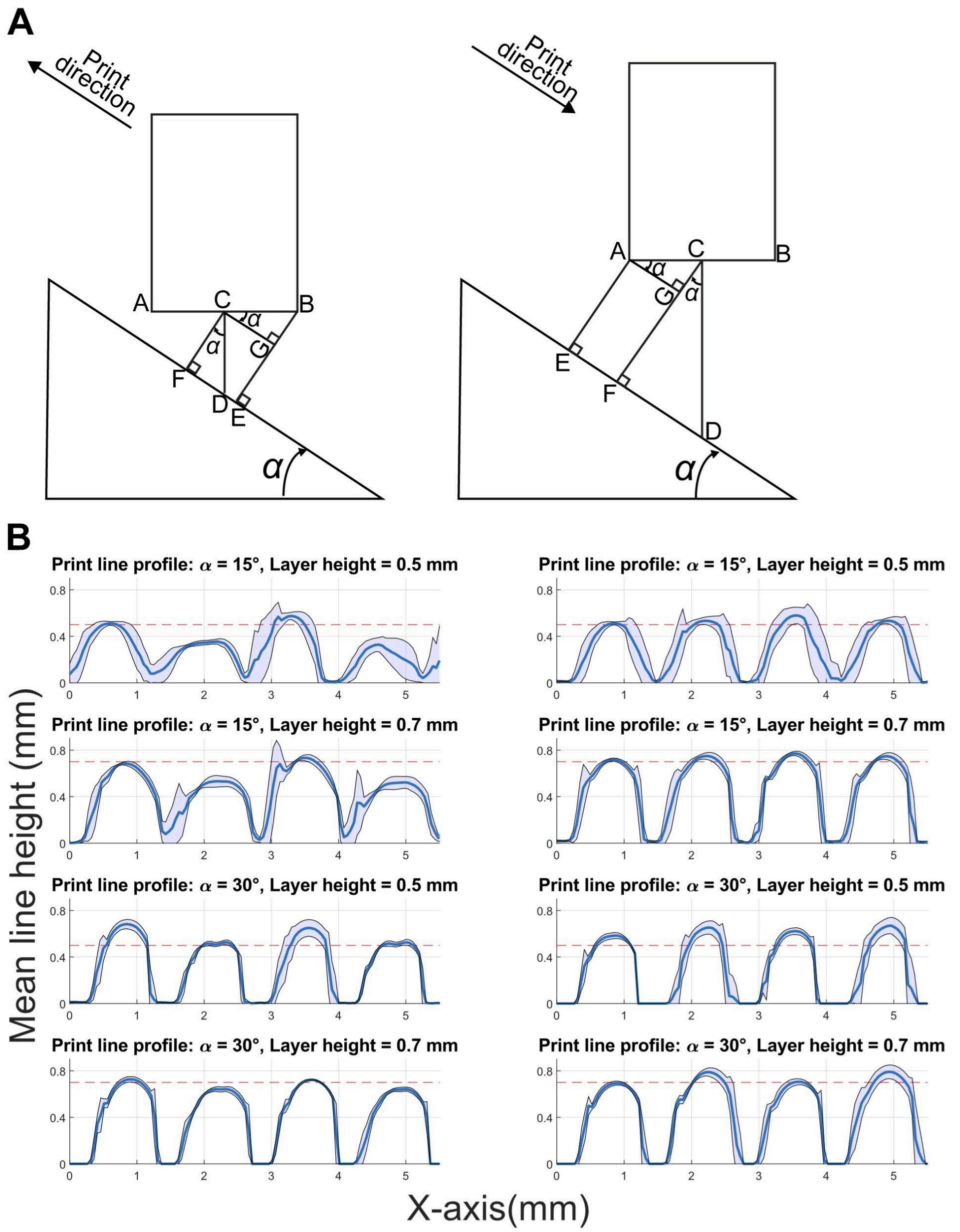

Figure S5. Deposition on inclined surfaces. (**A**) Geometrical position of the nozzle relative to the substrate when moving up (left) and down (right) an inclined plane for accurate deposition. (**B**) Lines were deposited by traversing up and down a fixed inclined plane and scanned using a laser displacement sensor. Cross-sectional profiles of deposited lines on inclined plane with no adjustment for gradient (i.e. $h = \frac{h_0}{\cos\alpha}$) are shown on the left and cross-sectional profiles after adjusting for gradient (i.e. $h = \frac{h_0}{\cos\alpha} \pm \left(\frac{d}{2}\right) * \tan\alpha$) are shown on the right. Cross-sectional profiles of the deposited lines are shown in blue while the expected print height is shown in red. The spread indicates standard deviation (n = 20). The cross-sectional profiles of lines deposited after accounting for the gradient are more uniform compared to the profiles on the left. Ink used was PD:RTVS=0.0 & FS=0.

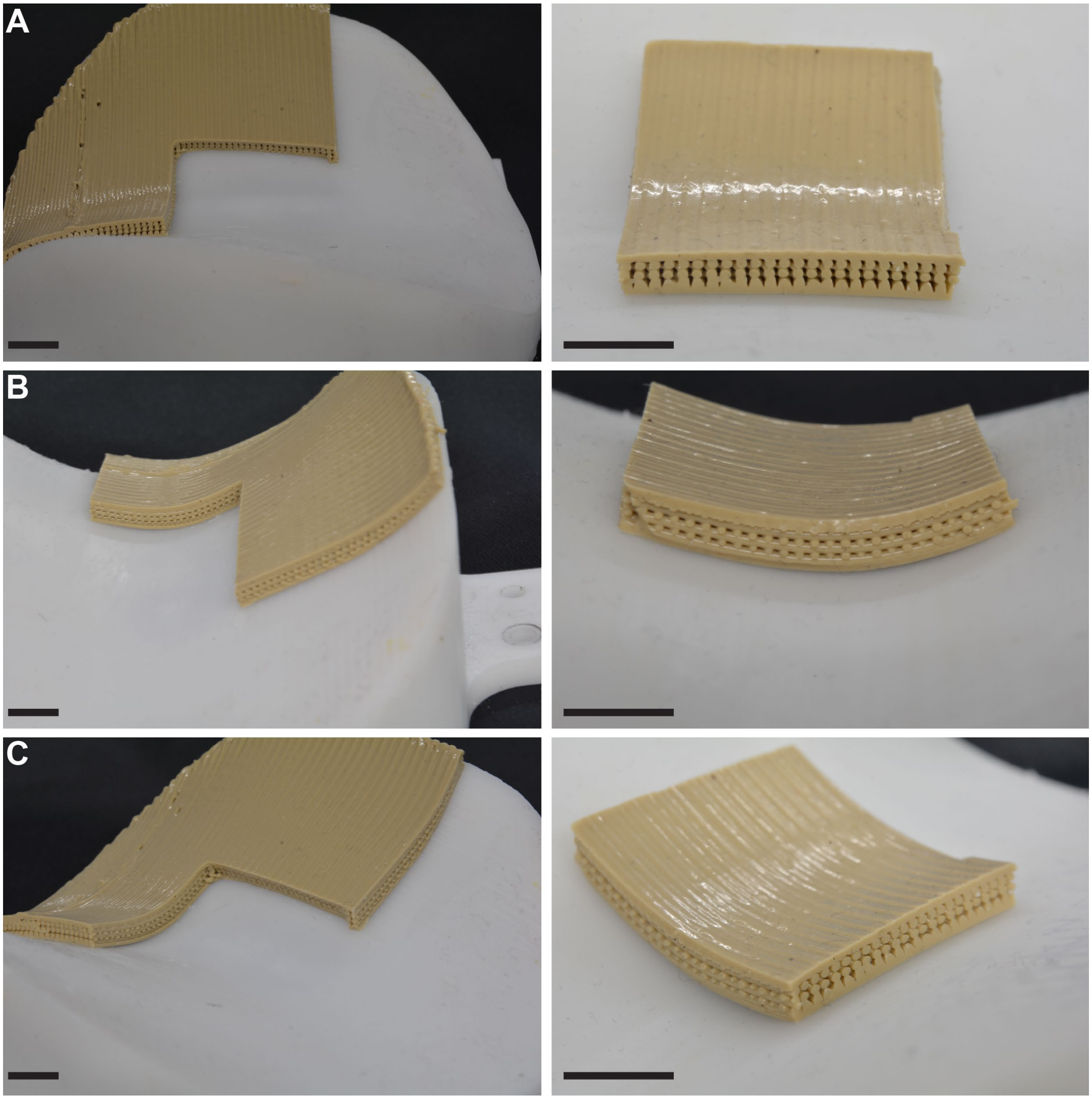

Figure S6. Cross-sectional images of the skin-layer of the 3D printed cric-skin puck. The images illustrate the accuracy of formation of the 3D printed cellular infill structure on non-planar substrates. (**A**) Cross-sectional view of print lines aligned in the longitudinal direction (Scale bar = 10 mm (accurate for foreground)). (**B**) Cross-sectional view of print lines aligned in the transverse direction (Scale bar = 10 mm (accurate for foreground)). (**C**) Angled view of print lines (Scale bar = 10 mm (applies at the mid-plane).

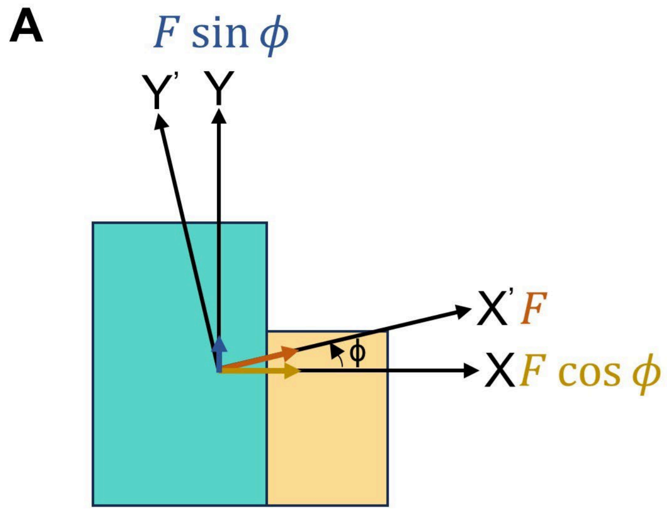

Figure S7. Variation in anisotropy with change in orientation relative to measurement axes. (**A**) Applied force in the longitudinal direction (X') oriented at an angle ϕ relative to orthogonal print-line directions (X-Y). (**B**) Applied force in the transverse direction (Y') oriented at an angle ϕ relative to orthogonal print-line directions (X-Y).

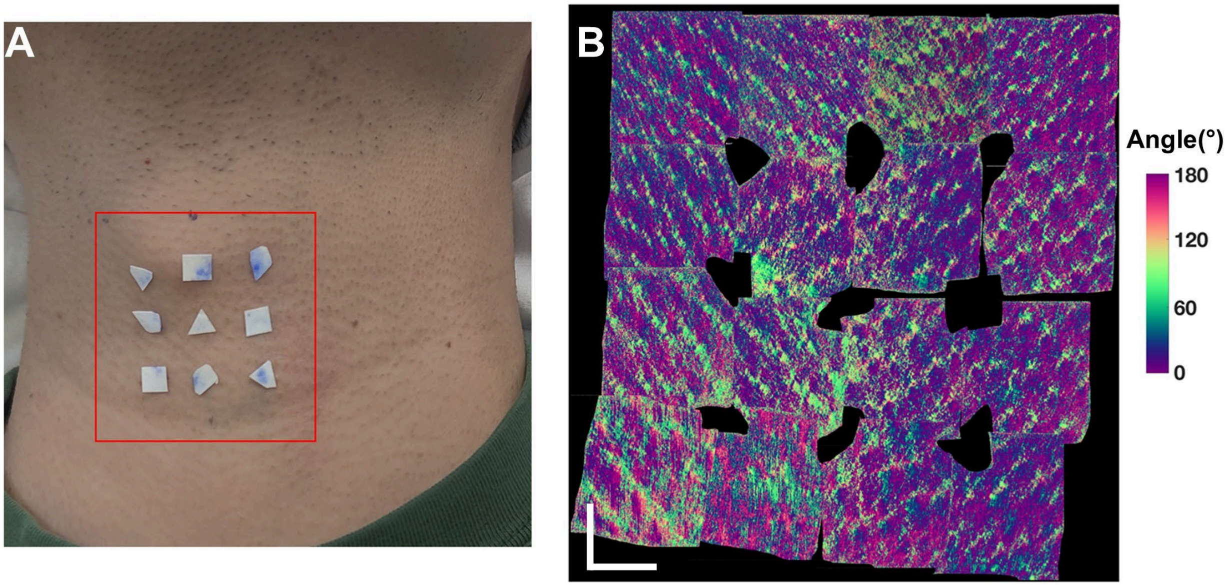

Figure S8. Identifying underlying collagen fiber orientations in tissue. (**A**) A photograph showing a region of the neck of the subject. The red box in the photograph is the total field of view of interest. Utilizing paper cutouts of varying shapes facilitated the computational stitching of the PSOCT result. (**B**) Polarization-sensitive optical coherence tomography (PSOCT) was used to map the fiber orientations at various locations. Figure shows the local optics axis of the red box region after computational stitching of 16 individual PSOCT scans. Apart from the hair follicle region, the collagen network appears to have a horizontal organization (Scale bar = 5000 μm).

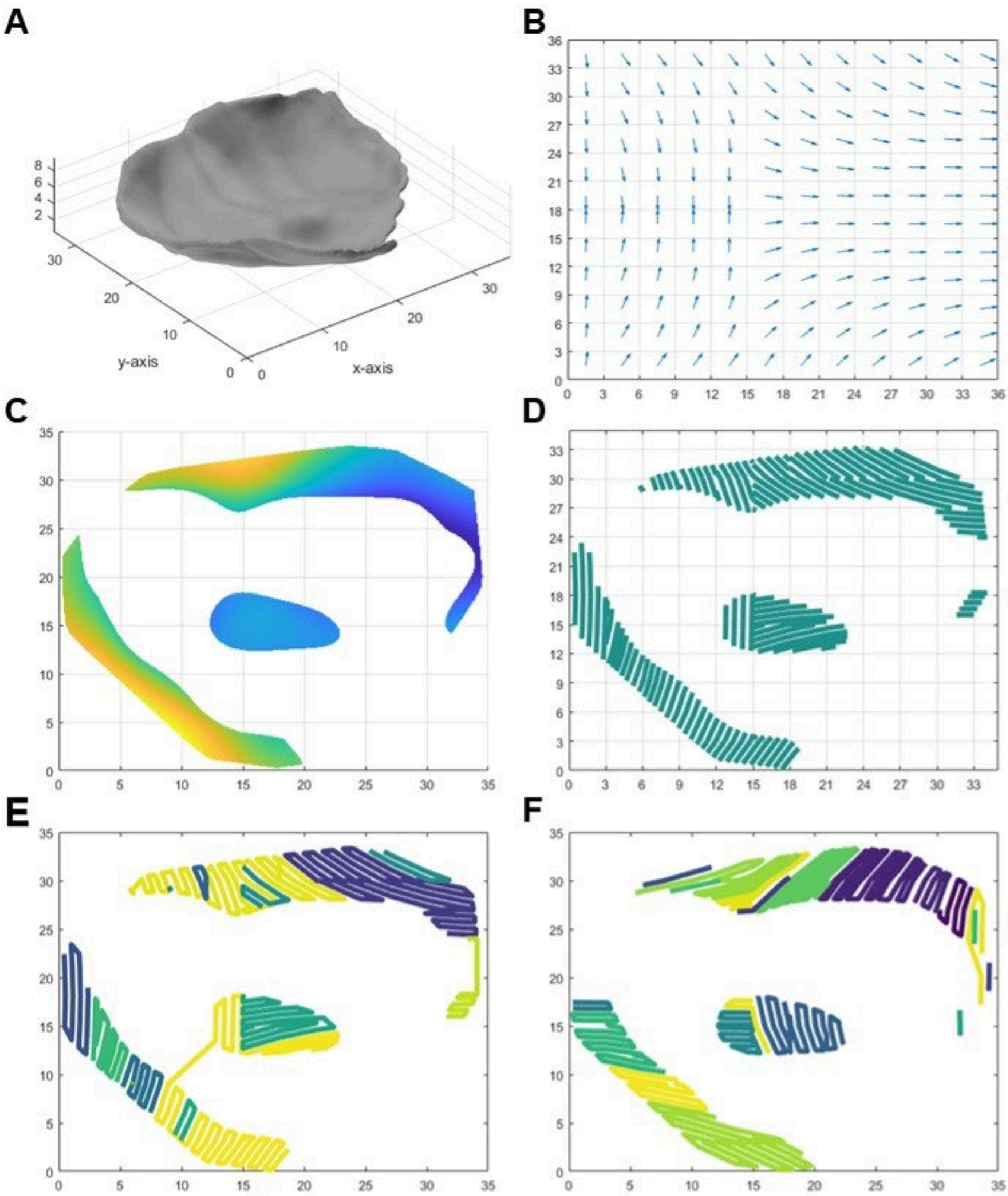

Figure S9. Process of generating toolpaths for a particular layer of a 3D printed heart valve model with local variations in directionality. (**A**) CAD model of a heart valve. (**B**) Input directions for local fiber orientations. (**C**) A typical infill layer of the heart valve. As the thickness is non-uniform, the layer has regions with and without any material. (**D**) Print-lines generated in accordance with local fiber orientation and slice boundaries. (**E-F**) Generated tool paths for printing the heart valve model with the desired anisotropy and directionality. Each color represents an individual toolpath. Alternate layers will have toolpaths that are orthogonal to one another.

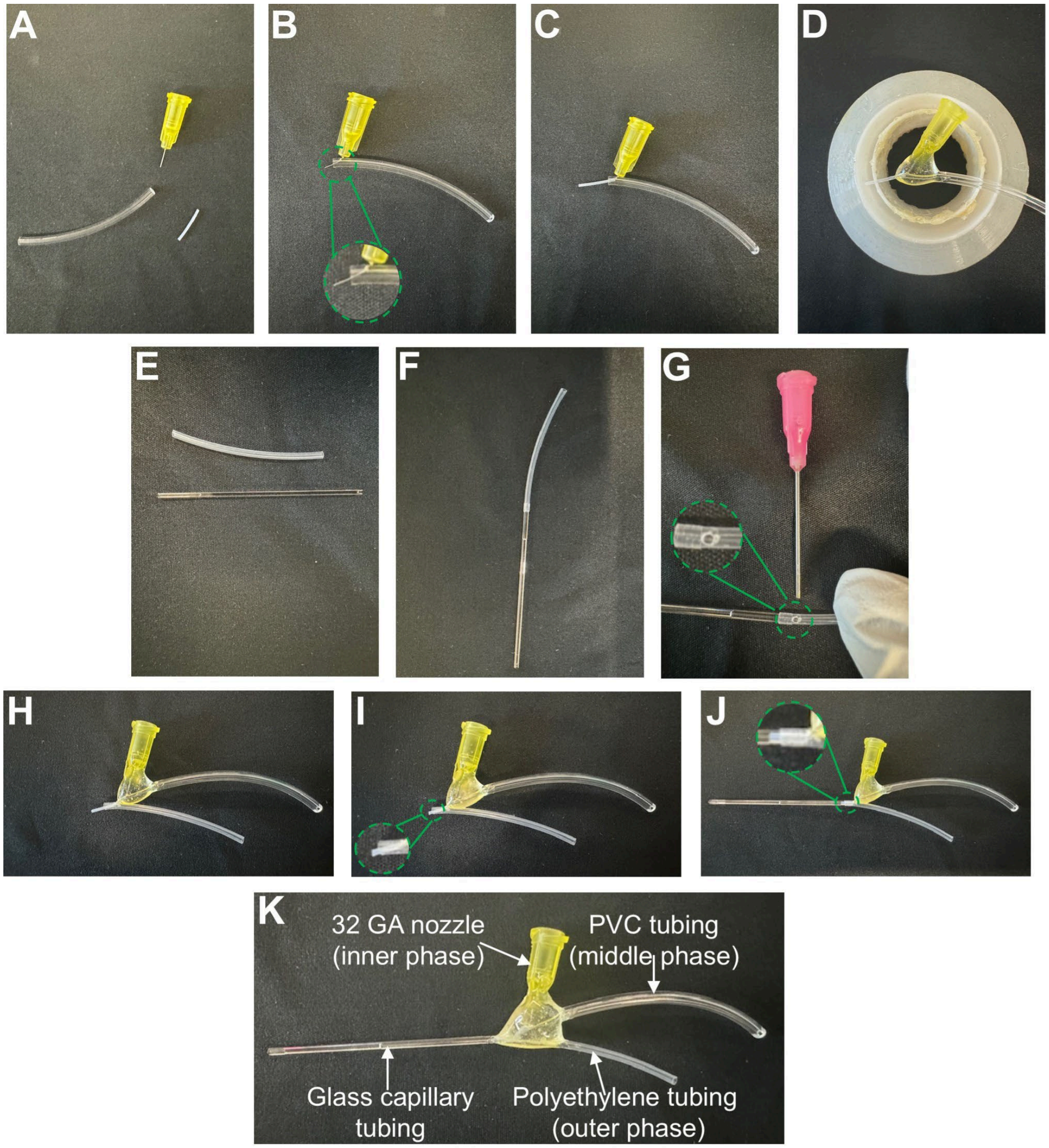

Figure S10. Development of the microfluidic chip for fluid-filled capsule production. (**A**) PVC and Teflon tubing were cut to desired lengths. (**B**) A 32 GA nozzle was poked through the top wall of the PVC tubing and inserted at an angle. (**C**) Teflon was inserted as a sheath over the 32 GA nozzle and into the PVC tubing. (**D**) Epoxy was then added to seal the Teflon-PVC-32 GA nozzle connection. (**E**) Polyethylene (PE) tubing was cut to the desired length, and a hydrophilic-treated glass capillary tube was introduced. (**F**) The capillary tubing was then inserted into the PE tubing. (**G**) A heated 20 GA nozzle was used to poke a hole in the PE tubing adjacent to the glass insertion point. (**H**) The glass tubing was removed, and the teflon-PVC-32 GA complex was inserted into the PE. (**I**) The Teflon was cut to minimally extend out of the PE tubing. (**J**) Glass capillary tubing was reinserted into the PE tubing and outside of the Teflon tubing. (**K**) The glass capillary tube-PE-Teflon connection was sealed with epoxy to create the final microfluidic chip.

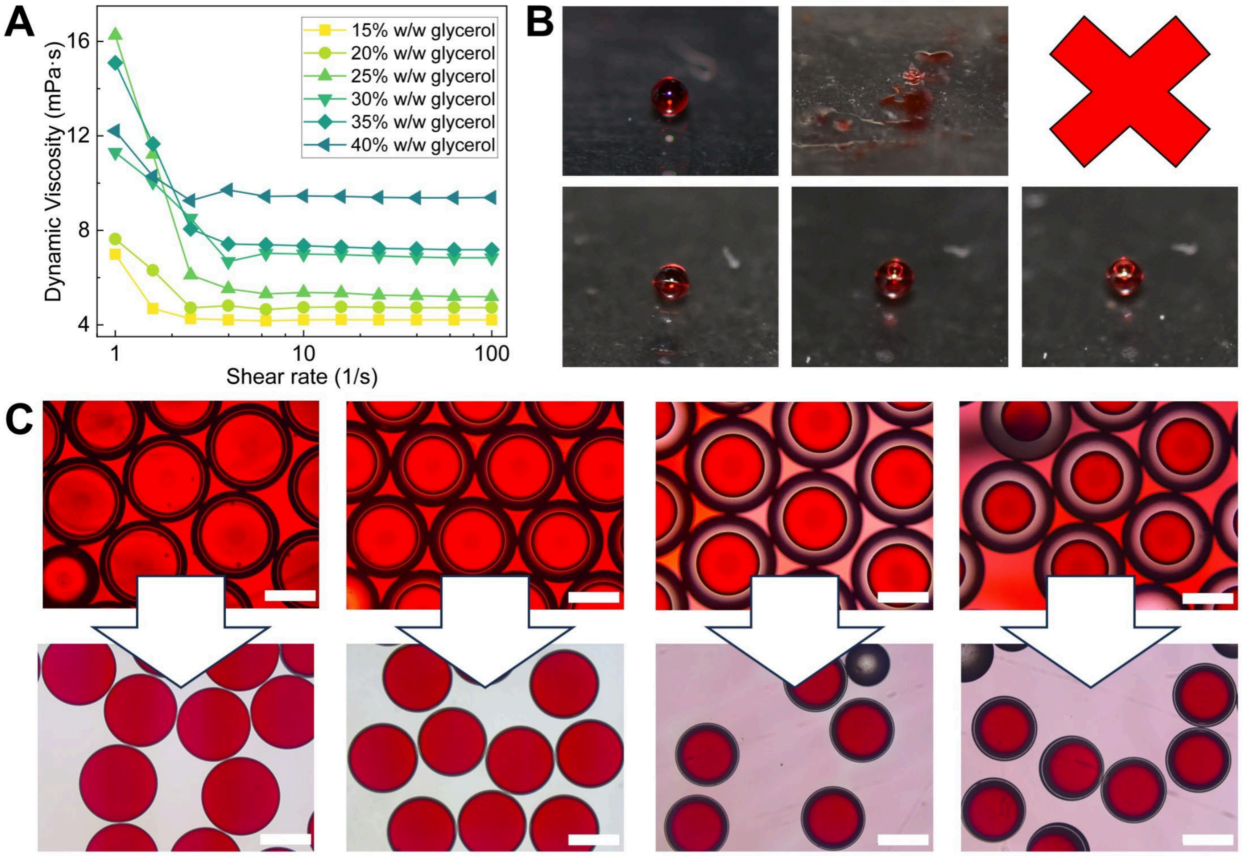

Figure S11. Optimization and customization of fluid-filled capsules. (**A**) Dynamic viscosities of capsule core solutions at 23 °C containing various glycerol concentrations. The dynamic viscosity of blood at 23°C is approximately 4 mPa·s at higher shear rates. (**B**) Open air stability testing of capsules with cores comprised of 15% w/w glycerol (top) compared with a 25% w/w glycerol (bottom). Images of capsules resting dry on glass slides were taken after 0, 24, and 48 hours (left, middle, right) unless the capsule had previously ruptured due to the internal vapor pressure. (**C**) Images of capsules produced with inner:middle phase flow rate ratios of 1:1, 1:2, 1:3, and 1:4 (left to right) immediately after fabrication (top) and after middle phase solvent evaporation (bottom). (Scale bar = 500 µm).

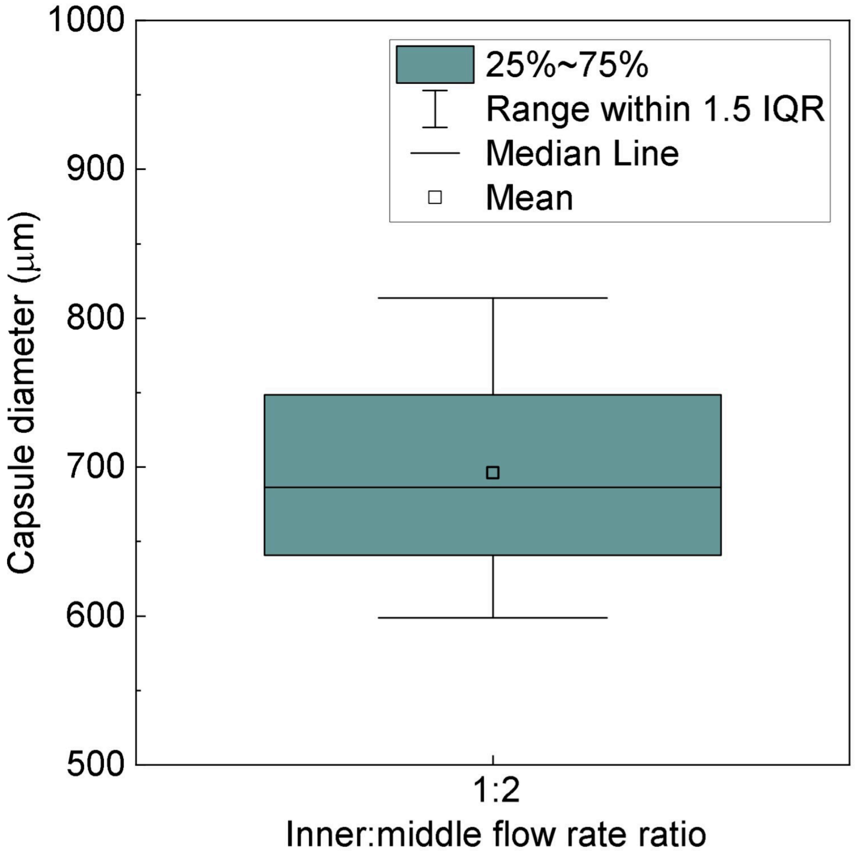

%tile	Size (μm)
10.00	598.8
20.00	622.0
30.00	640.7
40.00	658.7
50.00	676.5
60.00	696.1
70.00	719.6
80.00	748.6
90.00	786.9
95.00	813.4

Fig. S12. Size distribution of fluid-filled capsules. Capsules were manufactured with a 1:2 ratio of inner:middle phase flow rate. The capsule size distribution median by volume was at a diameter of 676.5 μm, as shown both via box chart (left) and table breakdown of percentiles (right). A similar value was found for the average capsule diameter measured from compression testing (673.6 ± 19 μm, n = 7).

Question	Cast (0-100)	3D printed (0-100)
1. Please indicate how closely does the simulated skin visually resemble real human skin?	67.92 ± 17.72[1]	60.15 ± 18.78[1]
2. Please indicate how closely does the subcutaneous tissue visually resemble real human tissue?	62.62 ± 15.14[1]	66.62 ± 11.08[1]
3. Please indicate how closely does the cricothyroid membrane visually resemble real human tissue?	61.69 ± 20.75[1]	68.62 ± 12.12[1]
**4. Please indicate how closely the simulated skin feels like the skin on a real human's neck while palpating**	**65 ± 40.25[2*]**	**73.5 ± 16.25[2*]**
5. Please indicate how closely the simulated subcutaneous tissue feels like a real human's neck while palpating	60.85 ± 18.15[1]	66.54 ± 12.34[1]
6. Please indicate how closely the simulated puck pinches like the skin of a real human's neck.	65.38 ± 16.67[1]	66.62 ± 12.35[1]
7. Please indicate how closely the simulated skin slides like the skin of a real human's neck.	63 ± 18.25[2]	68.5 ± 13.50[2]
**8. Please indicate how closely the simulated skin bleeds like the skin of a real human's neck.**	**47.23 ± 31.65[1*]**	**74.77 ± 12.41[1*]**
**9. Please indicate how closely the simulated skin cuts like the skin of a real human's neck.**	**63 ± 25.00[2*]**	**69 ± 14.75[2*]**
**10. Please indicate how difficult it was to recover a lost incision site when attempting to place an endotracheal tube through the cricothyroid membrane.**	**48.5 ± 29.50 [2*]**	**63.50 ± 27.75[2*]**
**11. On a scale of 0 to 100, with 0 = not at all acceptable for training and 100 = Ideal for training, please indicate your preference for using each puck to train the skills required to perform a cricothyrotomy.**	**75.92 ± 11.77[1*]**	**84.25 ± 11.10[1*]**

[1]mean ± std [2]median ± IQR [*]Statistically significant ($p<0.05$)

Table S1. Complete survey of questions and responses from comparative acceptability study. A paired samples one-sided t-test was used to evaluate our hypothesis. Questions 4, 7, 9 and 10 were found to have outliers present in the data. In that case, a Wilcoxon signed rank test was used to evaluate significance.

Movie S1. 3D printing cric-skin puck infill with local variations in directionality.

Movie S2. 3D printing aortic valve model with rectangular anisotropy.

Movie S3. Fluid-filled capsule production.

Movie S4. 3D printing cric-skin puck with embedded blood-simulating capsules.

Movie S5. Cric-skin puck bleeding demo.

Movie S6. Comparative acceptability study.

REFERENCES AND NOTES

1. R. J. Scalese, V. T. Obeso, S. B. Issenberg, Simulation technology for skills training and competency assessment in medical education. *J. Gen. Intern. Med.* **23**, 46–49 (2008).

2. H. Owen, Early use of simulation in medical education. *Simul. Healthc.* **7**, 102–116 (2012).

3. N. J. Maran, R. J. Glavin, Low- to high-fidelity simulation – A continuum of medical education. *Med. Educ.* **37** (Suppl. 1), 22–28 (2003).

4. S. Maglio, C. Park, S. Tognarelli, A. Menciassi, E. T. Roche, High-fidelity physical organ simulators: From artificial to bio-hybrid solutions. *IEEE Trans. Med. Robot. Bionics* **3**, 349–361 (2021).

5. M. Mulyadi, S. I. Tonapa, S. S. J. Rompas, R.-H. Wang, B.-O. Lee, Effects of simulation technology-based learning on nursing students' learning outcomes: A systematic review and meta-analysis of experimental studies. *Nurse Educ. Today* **107**, 105127 (2021).

6. C. Elendu, D. C. Amaechi, A. U. Okatta, E. C. Amaechi, T. C. Elendu, C. P. Ezeh, I. D. Elendu, The impact of simulation-based training in medical education: A review. *Medicine* **103**, e38813 (2024).

7. A. Haiser, A. Aydin, B. Kunduzi, K. Ahmed, P. Dasgupta, A systematic review of simulation-based training in vascular surgery. *J. Surg. Res.* **279**, 409–419 (2022).

8. T. R. Meling, T. R. Meling, The impact of surgical simulation on patient outcomes: A systematic review and meta-analysis. *Neurosurg. Rev.* **44**, 843–854 (2021).

9. J. F. Uhl, A. Sufianov, C. Ruiz, Y. Iakimov, H. J. Mogorron, M. Encarnacion Ramirez, G. Prat, B. Lorea, M. Baldoncini, E. Goncharov, I. Ramirez, J. R. C. Céspedes, R. Nurmukhametov, N. Montemurro, The use of 3D printed models for surgical simulation of cranioplasty in craniosynostosis as training and education. *Brain Sci.* **13**, 894 (2023).

10. N. Wake, H. Chandarana, W. C. Huang, S. S. Taneja, A. B. Rosenkrantz, Application of anatomically accurate, patient-specific 3D printed models from MRI data in urological oncology. *Clin. Radiol.* **71**, 610–614 (2016).

11. J.-C. Bernhard, S. Isotani, T. Matsugasumi, V. Duddalwar, A. J. Hung, E. Suer, E. Baco, R. Satkunasivam, H. Djaladat, C. Metcalfe, B. Hu, K. Wong, D. Park, M. Nguyen, D. Hwang, S. T. Bazargani, A. L. de Castro Abreu, M. Aron, O. Ukimura, I. S. Gill, Personalized 3D printed model of kidney and tumor anatomy: A useful tool for patient education. *World J. Urol.* **34**, 337–345 (2016).

12. K. Qiu, Z. Zhao, G. Haghiashtiani, S.-Z. Guo, M. He, R. Su, Z. Zhu, D. B. Bhuiyan, P. Murugan, F. Meng, S. H. Park, C.-C. Chu, B. M. Ogle, D. A. Saltzman, B. R. Konety, R. M. Sweet, M. C. McAlpine, 3D printed organ models with physical properties of tissue and integrated sensors. *Adv. Mater. Technol.* **3**, 1700235 (2018).

13. K. Qiu, G. Haghiashtiani, M. C. McAlpine, 3D printed organ models for surgical applications. *Annu. Rev. Anal. Chem.* **11**, 287–306 (2018).

14. G. Haghiashtiani, K. Qiu, J. D. Zhingre Sanchez, Z. J. Fuenning, P. Nair, S. E. Ahlberg, P. A. Iaizzo, M. C. McAlpine, 3D printed patient-specific aortic root models with internal sensors for minimally invasive applications. *Sci. Adv.* **6**, eabb4641 (2020).

15. J. Li, X. Y. Luo, Z. B. Kuang, A nonlinear anisotropic model for porcine aortic heart valves. *J. Biomech.* **11**, 1279–1289 (2001).

16. C. Storm, J. J. Pastore, F. C. MacKintosh, T. C. Lubensky, P. A. Janmey, Nonlinear elasticity in biological gels. *Nature* **435**, 191–194 (2005).

17. A. Ni Annaidh, K. Bruyère, M. Destrade, M. D. Gilchrist, M. Otténio, Characterization of the anisotropic mechanical properties of excised human skin. *J. Mech. Behav. Biomed. Mater.* **5**, 139–148 (2012).

18. Y. Feng, R. J. Okamoto, R. Namani, G. M. Genin, P. V. Bayly, Measurements of mechanical anisotropy in brain tissue and implications for transversely isotropic material models of white matter. *J. Mech. Behav. Biomed. Mater.* **23**, 117–132 (2013).

19. K. B. Gupta, M. B. Ratcliffe, M. A. Fallert, L. H. Edmunds, D. K. Bogen, Changes in passive mechanical stiffness of myocardial tissue with aneurysm formation. *Circulation* **89**, 2315–2326 (1994).

20. J. A. Stella, J. Liao, M. S. Sacks, Time-dependent biaxial mechanical behavior of the aortic heart valve leaflet. *J. Biomech.* **40**, 3169–3177 (2007).

21. A. Joda, Z. Jin, A. Haverich, J. Summers, S. Korossis, Multiphysics simulation of the effect of leaflet thickness inhomogeneity and material anisotropy on the stress–strain distribution on the aortic valve. *J. Biomech.* **49**, 2502–2512 (2016).

22. C. Schumacher, B. Bickel, J. Rys, S. Marschner, C. Daraio, M. Gross, Microstructures to control elasticity in 3D printing. *ACM Trans. Graph.* **34**, 1–13 (2015).

23. S. Makode, G. Singh, A. Chanda, Development of novel anisotropic skin simulants. *Phys. Scr.* **96**, 125019 (2021).

24. V. Gupta, R. Singla, G. Singh, A. Chanda, Development of soft composite based anisotropic synthetic skin for biomechanical testing. *Fibers* **11**, 55 (2023).

25. B. G. Compton, J. A. Lewis, 3D-printing of lightweight cellular composites. *Adv. Mater.* **26**, 5930–5935 (2014).

26. J. L. Apfelbaum, C. A. Hagberg, R. T. Connis, B. B. Abdelmalak, M. Agarkar, R. P. Dutton, J. E. Fiadjoe, R. Greif, P. A. Klock Jr., D. Mercier, S. N. Myatra, E. P. O'Sullivan, W. H. Rosenblatt, M. Sorbello, A. Tung, 2022 American Society of Anesthesiologists practice guidelines for management of the difficult airway. *Anesthesiology* **136**, 31–81 (2022).

27. L. Gaitini, M. Á. Gómez-Ríos, O. Hochman, P. Charco-Mora, M. Somri, Cricothyrotomy in difficult airway management: A narrative review. *Trends Anaesth. Crit. Care* **50**, 101249 (2023).

28. C. A. Brown, A. Fantegrossi, O. Baker, R. M. Walls, Decline in rates of cricothyrotomy in the emergency department. *Ann. Emerg. Med.* **74**, S16 (2019).

29. M. B. Blackburn, M. D. April, D. J. Brown, R. A. DeLorenzo, K. L. Ryan, A. N. Blackburn, S. G. Schauer, Prehospital airway procedures performed in trauma patients by ground forces in Afghanistan. *J. Trauma Acute Care Surg.* **85**, S154–S160 (2018).

30. A. L. Makowski, A survey of graduating emergency medicine residents' experience with cricothyrotomy. *West. J. Emerg. Med.* **14**, 654–661 (2013).

31. B. Backlund, R. Utarnachitt, J. Jauregui, T. Watase, A comprehensive course for teaching emergency cricothyrotomy. *J. Educ. Teach. Emerg. Med.* **5**, SG17–SG35 (2020).

32. J. M. Aho, C. A. Thiels, Y. N. AlJamal, R. K. Ruparel, P. G. Rowse, S. F. Heller, D. R. Farley, Every surgical resident should know how to perform a cricothyrotomy: An inexpensive cricothyrotomy task trainer for teaching and assessing surgical trainees. *J. Surg. Educ.* **72**, 658–661 (2015).

33. D. Hart, R. Rush, G. Rule, J. Clinton, G. Beilman, S. Anders, R. Brown, M. A. McNeil, T. Reihsen, J. Chipman, R. Sweet, University of Minnesota Combat Casualty Training Consortium (UMN CCTC), Training and assessing critical airway, breathing, and hemorrhage control procedures for trauma care: Live tissue versus synthetic models. *Acad. Emerg. Med.* **25**, 148–167 (2018).

34. J. K. Takayesu, D. Peak, D. Stearns, Cadaver-based training is superior to simulation training for cricothyrotomy and tube thoracostomy. *Intern. Emerg. Med.* **12**, 99–102 (2017).

35. K. E. Hughes, D. Biffar, E. O. Ahanonu, T. M. Cahir, A. Hamilton, J. C. Sakles, Evaluation of an innovative bleeding cricothyrotomy model. *Cureus* **10**, e3327 (2018).

36. S. F. Miller, J. Sanz-Guerrero, R. E. Dodde, D. D. Johnson, A. Bhawuk, H. S. Gurm, A. J. Shih, A pulsatile blood vessel system for a femoral arterial access clinical simulation model. *Med. Eng. Phys.* **35**, 1518–1524 (2013).

37. A. M. DeSchmidt, A. T. Gong, J. E. Batista, A. Y. Song, S. L. Bidinger, A. L. Schul, E. Y. Wang, J. E. Norfleet, R. M. Sweet, Characterization of puncture forces of the human trachea and cricothyroid membrane. *J. Biomech. Eng.* **144**, 104502 (2022).

38. J. E. Smay, J. Cesarano, J. A. Lewis, Colloidal inks for directed assembly of 3-D periodic structures. *Langmuir* **18**, 5429–5437 (2002).

39. Y. Wang, N. Willenbacher, Phase-change-enabled, rapid, high-resolution direct ink writing of soft silicone. *Adv. Mater.* **34**, 2109240 (2022).

40. V. A. Roach, D. J. Traina, D. M. Hananel, J. R. Speich, J. E. Norfleet, R. M. Sweet, Development and evaluation of the Advanced Joint Airway Management System for educational utility in endotracheal intubation, as assessed by expert paramedic instructors. *Mil. Med.* **189**, 702–709 (2024).

41. J. E. Smay, G. M. Gratson, R. F. Shepherd, J. Cesarano III, J. A. Lewis, Directed colloidal assembly of 3D periodic structures. *Adv. Mater.* **14**, 1279–1283 (2002).

42. F. Khatyr, C. Imberdis, P. Vescovo, D. Varchon, J.-M. Lagarde, Model of the viscoelastic behaviour of skin in vivo and study of anisotropy. *Skin Res. Technol.* **10**, 96–103 (2004).

43. J. A. Lewis, Direct-write assembly of ceramics from colloidal inks. *Curr. Opin. Solid State Mater. Sci.* **6**, 245–250 (2002).

44. G. Q. Zhang, W. Mondesir, C. Martinez, X. Li, T. A. Fuhlbrigge, H. Bheda, "Robotic additive manufacturing along curved surface — A step towards free-form fabrication," in *2015 IEEE International Conference on Robotics and Biomimetics (ROBIO)* (IEEE, 2015), pp. 721–726. https://ieeexplore.ieee.org/abstract/document/7418854.

45. C. D. Armstrong, S. M. Montgomery, L. Yue, F. Demoly, K. Zhou, H. J. Qi, Robotic conformal material extrusion 3D printing for appending structures on unstructured surfaces. *Adv. Intell. Syst.* **6**, 2300516 (2024).

46. F. Hong, S. Hodges, C. Myant, D. E. Boyle, "Open5x: Accessible 5-axis 3D printing and conformal slicing," in *Extended Abstracts of the 2022 CHI Conference on Human Factors in*

Computing Systems (CHI EA '22) (Association for Computing Machinery, 2022), pp. 1–6. https://dl.acm.org/doi/10.1145/3491101.3519782.

47. M. Vatani, F. Alkadi, J.-W. Choi, Algorithm to reduce leading and lagging in conformal direct-print. *J. Manuf. Sci. Eng.* **140**, 101014 (2018).

48. S. G. M. Uzel, R. D. Weeks, M. Eriksson, D. Kokkinis, J. A. Lewis, Multimaterial multinozzle adaptive 3D printing of soft materials. *Adv. Mater. Technol.* 2101710 (2022).

49. B. de Campos Vidal, M. L. S. Mello, Optical anisotropy of collagen fibers of rat calcaneal tendons: An approach to spatially resolved supramolecular organization. *Acta Histochem.* **112**, 53–61 (2010).

50. M. Zaffar, A. Pradhan, Assessment of anisotropy of collagen structures through spatial frequencies of Mueller matrix images for cervical pre-cancer detection. *Appl. Optics* **59**, 1237–1248 (2020).

51. S. K. de Visser, J. C. Bowden, E. Wentrup-Byrne, L. Rintoul, T. Bostrom, J. M. Pope, K. I. Momot, Anisotropy of collagen fibre alignment in bovine cartilage: Comparison of polarised light microscopy and spatially resolved diffusion-tensor measurements. *Osteoarthr. Cartil.* **16**, 689–697 (2008).

52. P. Tang, M. A. Kirby, N. Le, Y. Li, N. Zeinstra, G. N. Lu, C. E. Murry, Y. Zheng, R. K. Wang, Polarization sensitive optical coherence tomography with single input for imaging depth-resolved collagen organizations. *Light Sci. Appl.* **10**, 237 (2021).

53. P. Tang, R. K. Wang, Polarization state tracing method to map local birefringent properties in samples using polarization sensitive optical coherence tomography. *Biomed. Opt. Express* **11**, 6852–6863 (2020).

54. On the anatomy and physiology of the skin I. The cleavability of the cutis. (Translated from Langer, K. (1861). Zur Anatomie und Physiologie der Haut. I. Uber die Spaltbarkeit der Cutis. Sitzungsbericht der Mathematisch-naturwissenschaftlichen Classe der Kaiserlichen Academie der Wissenschaften, 44, 19.). *Br. J. Plast. Surg.* **31**, 3–8 (1978).

55. D. Chong, X. Liu, H. Ma, G. Huang, Y. L. Han, X. Cui, J. Yan, F. Xu, Advances in fabricating double-emulsion droplets and their biomedical applications. *Microfluid. Nanofluidics* **19**, 1071–1090 (2015).

56. L. Zhao-Miao, D. Yu, P. Yan, Generation of water-in-oil-in-water (W/O/W) double emulsions by microfluidics. *Chin. J. Anal. Chem.* **46**, 324–330 (2018).

57. M. Villiger, E. Z. Zhang, S. K. Nadkarni, W.-Y. Oh, B. J. Vakoc, B. E. Bouma, Spectral binning for mitigation of polarization mode dispersion artifacts in catheter-based optical frequency domain imaging. *Opt. Express* **21**, 16353–16369 (2013).
